# Fecal ^1^H-NMR Metabolomics: A Comparison of Sample Preparation Methods for NMR and Novel in Silico Baseline Correction

**DOI:** 10.3390/metabo12020148

**Published:** 2022-02-05

**Authors:** Catherine L. J. Brown, Hannah Scott, Crystal Mulik, Amy S. Freund, Michael P. Opyr, Gerlinde A. S. Metz, G. Douglas Inglis, Tony Montina

**Affiliations:** 1 Agriculture and Agri-Food Canada, Lethbridge Research and Development Centre, 5403-1st Avenue S, Lethbridge, AB T1J 4B1, Canada; kate.brown@uleth.ca; 2 Department of Biological Sciences, University of Lethbridge, 4401 University Drive, Lethbridge, AB T1K 3M4, Canada; 3 Southern Alberta Genome Sciences Centre, University of Lethbridge, Lethbridge, AB T1K 3M4, Canada; hscott@mitacs.ca (H.S.); crystal.mulik@uleth.ca (C.M.); 4 Canadian Centre for Behavioural Neuroscience, University of Lethbridge, Lethbridge, AB T1K 3M4, Canada; 5 Department of Neuroscience, University of Lethbridge, Lethbridge, AB T1K 3M4, Canada; 6 Department of Chemistry and Biochemistry, University of Lethbridge, Lethbridge, AB T1K 3M4, Canada; mikeopyr@gmail.com; 7 Bruker BioSpin Corporation, 19 Fortune Drive, Billerica, MA 01821, USA; Amy.Freund@bruker.com

**Keywords:** ^1^H-NMR, metabolomics, feces, intestine, enteric, microbiome, rats, chickens

## Abstract

Analysis of enteric microbiota function indirectly through the fecal metabolome has the potential to be an informative diagnostic tool. However, metabolomic analysis of feces is hampered by high concentrations of macromolecules such as proteins, fats, and fiber in samples. Three methods—ultrafiltration (UF), Bligh–Dyer (BD), and no extraction (samples added directly to buffer, vortexed, and centrifuged)—were tested on multiple rat (*n* = 10) and chicken (*n* = 8) fecal samples to ascertain whether the methods worked equally well across species and individuals. An in silico baseline correction method was evaluated to determine if an algorithm could produce spectra similar to those obtained via UF. For both rat and chicken feces, UF removed all macromolecules and produced no baseline distortion among samples. By contrast, the BD and no extraction methods did not remove all the macromolecules and produced baseline distortions. The application of in silico baseline correction produced spectra comparable to UF spectra. In the case of no extraction, more intense peaks were produced. This suggests that baseline correction may be a cost-effective method for metabolomic analyses of fecal samples and an alternative to UF. UF was the most versatile and efficient extraction method; however, BD and no extraction followed by baseline correction can produce comparable results.

## 1. Introduction

The enteric microbiota plays a critical role in the health of mammals and avians, and the structure and function of the microbiota can be an indicator of the health status of the host. The enteric microbiota impacts host health in a number of ways, including digestion of foods (e.g., fermentation of plant fiber), production of short-chain fatty acids and vitamins, stimulation and modulation of immune responses, and protection of the host from pathogens, to name a few [1,2]. Alterations to the normal structure of the microbiota (i.e., dysbiosis) are associated with a number of adverse acute and chronic health conditions such as pseudomembranous colitis [3,4], inflammatory bowel disease (IBD) [5], and irritable bowel syndrome (IBS) [6]. Autoimmune disorders such as multiple sclerosis (MS) [7], mood disorders such as major depressive disorder (MDD) [8], autism spectrum disorder (ASD) [9], and neurodegenerative diseases such as human motor neuron disease (MND) [10] have also been linked to dysbioses in the enteric microbiota. However, whether this is cause or effect is unclear, and the mechanisms by which the microbiome affects host health are currently enigmatic.

In recent years, an emphasis has been placed on characterizing the composition and structure of the enteric microbiota using 16S rRNA gene sequencing [11]. However, this technique does not directly measure bacterial functions such as metabolic processes [12]. Another disadvantage of 16S rRNA gene sequencing is that it currently delivers poor taxonomic resolution (i.e., family or genus), it is relatively time and bioinformatically intensive, and it can be cost-prohibitive [12,13]. Using metabolomics to ascertain the function of the microbiota, including the impact of enteric dysbioses on host health, has considerable benefits, and it is receiving increasing attention for applications in human and non-human medicine. Metabolomics, the study of the small molecular weight molecules forming and resulting from the biochemical pathways of an organism, can be used to characterize the metabolome in a variety of tissues and biofluids [14,15,16]. The fecal metabolome reflects host–microbiome interactions. However, up to 68% of its variance arises from the enteric microbiota, and not the host [17]. As the relationship between the enteric microbiome and health of the host becomes more evident, there is increasing interest in using fecal metabolomics as an indirect and non-invasive diagnostic indicator of host health.

Despite the presence of established methods and databases for proton nuclear magnetic resonance (^1^H-NMR)-based metabolomic analysis of serum [18], urine [19], and saliva [20], characterization of the fecal metabolome is an emerging field with a large amount of variation in sample preparation methods reported among studies [21,22,23,24,25]. The analysis of fecal samples using metabolomic methods presents a series of unique challenges, namely the solid nature of the sample and the presence of varying and large amounts of molecules such as water, proteins, fats, and fiber. Notably, inconsistent and incomplete removal of fats and macromolecules from fecal samples can result in an increase in the number of broad signals in the baseline of ^1^H-NMR spectra, and these signals can often span several parts per million on the NMR chemical shift scale. As a direct result of this, there is a great deal of inaccuracy in the quantification of any metabolite that overlaps with these broad signals [26].

Previous studies have examined the effects of various sample processing and metabolite extraction methods of feces to determine which are the most unbiased, efficient, and reproducible. These include, but are not limited to, fresh versus frozen fecal samples [27,28], pooled versus un-pooled samples [29], freeze-dried versus frozen samples [21,24,25,27,29,30,31,32], refrigerated versus frozen samples [33], different homogenization methods, and the ratio of feces to buffer (W_f_:V_b_) [34]. Although currently published studies present important information, there is currently no universal consensus on the best methods by which to prepare and analyze fecal samples for metabolomic analysis using ^1^H-NMR [35]. In this regard, Cui et al. [35] evaluated several different methods for preparation of fecal samples for ^1^H-NMR, including a comparison of extraction solvents and processing in terms of the reproducibility of signature metabolites. However, they did not test ultrafiltration (UF), which has been recommended for many types of samples [18,36]; UF provides good signal-to-noise, provides good reproducibility without unwanted evaporation of volatile metabolites, and avoids loss of metabolites by dissolution in solvents or the hydrolysis of metabolites due to the need to neutralize the solution [36]. An alternative approach for the consistent removal of broad signals from NMR spectra is in silico baseline correction. With respect to metabolomics, however, baseline correction has only been applied to smoothing or flattening distortions in the baseline of a spectrum, such as rolling baselines; it has not been applied in metabolomic studies to remove the large broad signals that may be present due to residual macromolecules [37,38], or has only been used as a component of a larger algorithm for quantification of in vivo spectra such as LCModel [39] and jMRUI [40].

The goal of the current study was to comparatively examine a number of sample preparation methods for fecal metabolomics analysis by ^1^H-NMR. The methods tested were: (1) no extraction, in which the fecal sample is added to buffer, vortexed, and centrifuged (hereafter referred to as “no extraction”); (2) UF [25]; (3) the traditional Bligh–Dyer (BD) fat and protein extraction method [34,41]; and (4) a modified version of the BD (M-BD) method that was intended for samples that have high fatty acid content [42]. Lastly, the most effective of these methods, UF, was compared to results achieved using both the no extraction and BD methods in which the spectra were processed using an in silico baseline correction. These methods were evaluated both qualitatively and quantitatively based on the least amount of baseline variation and the most consistent results across samples.

## 2. Results and Discussion

Quantifying changes in the fecal metabolome can provide valuable insights into the functioning of the enteric microbiota and interactions with the host. However, fecal samples present a unique challenge due to the varying amounts of water and macromolecules, such as proteins, fats, and fiber, that are present and interfere with quantification in NMR-based metabolomics. Currently, there are many different methods proposed for the preparation of fecal samples for ^1^H-NMR; however, there is no universal consensus on the most cost-effective and reproducible method for water-soluble metabolite extraction. Thus, an overarching goal of the current study was to investigate various sample preparation methods in order to determine the best way to effectively and reproducibly remove unwanted macromolecules while preserving the small molecule water-soluble metabolites [35]. We evaluated four different extraction methods as well as the application of in silico baseline correction.

### 2.1. Comparison of Different Metabolite Extraction Methods

**Figure 1:**
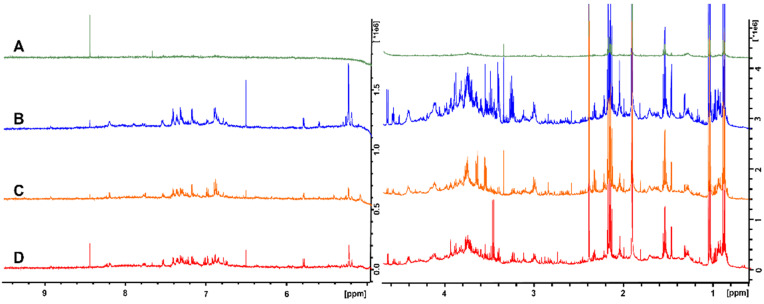
NMR spectra of rat fecal samples that were processed using four different techniques: (**A**) fatty acid favoring extraction; (**B**) no extraction; (**C**) ultrafiltration; and (**D**) Bligh–Dyer extraction. The spectra have been split at the water peak and the vertical scale has been increased to better illustrate the metabolites that are present.

The fatty acid favoring extraction method (M-BD) provided a spectrum with a greatly reduced number of peaks, and the signals were much less intense when compared to the other methods (Figure 1A). This was particularly evident for the peaks corresponding to glucose and succinic acid at ≈5.3 and 2.4 ppm, respectively. The only exception was the formic acid peak at 8.4 ppm, which was most intense when using the M-BD extraction. Overall, the M-BD extraction removed or reduced almost all of the metabolite peaks, and thus it was not pursued further. The peaks in the no extraction spectrum (Figure 1B) were more intense than the majority of the peaks in the BD extraction (Figure 1C) and UF (Figure 1D) spectra. For example, the argininosuccinic acid, glucose, and butyric acid peaks at 6.5 ppm, 5.3/4.7 ppm, and 1.6 ppm, respectively, were noticeably more intense. In addition, a conspicuous reduction in the spectral intensity of all metabolites was observed for the BD extraction method versus the no extraction and UF methods. The UF, no extraction, and BD extraction methods were all chosen for analysis of inter-sample variability, as in contrast to the M-BD method, all three contained sufficient metabolite information to conduct non-targeted metabolomic analyses.

### 2.2. Inter-Sample Variability and Baseline Distortion

**Figure 2:**
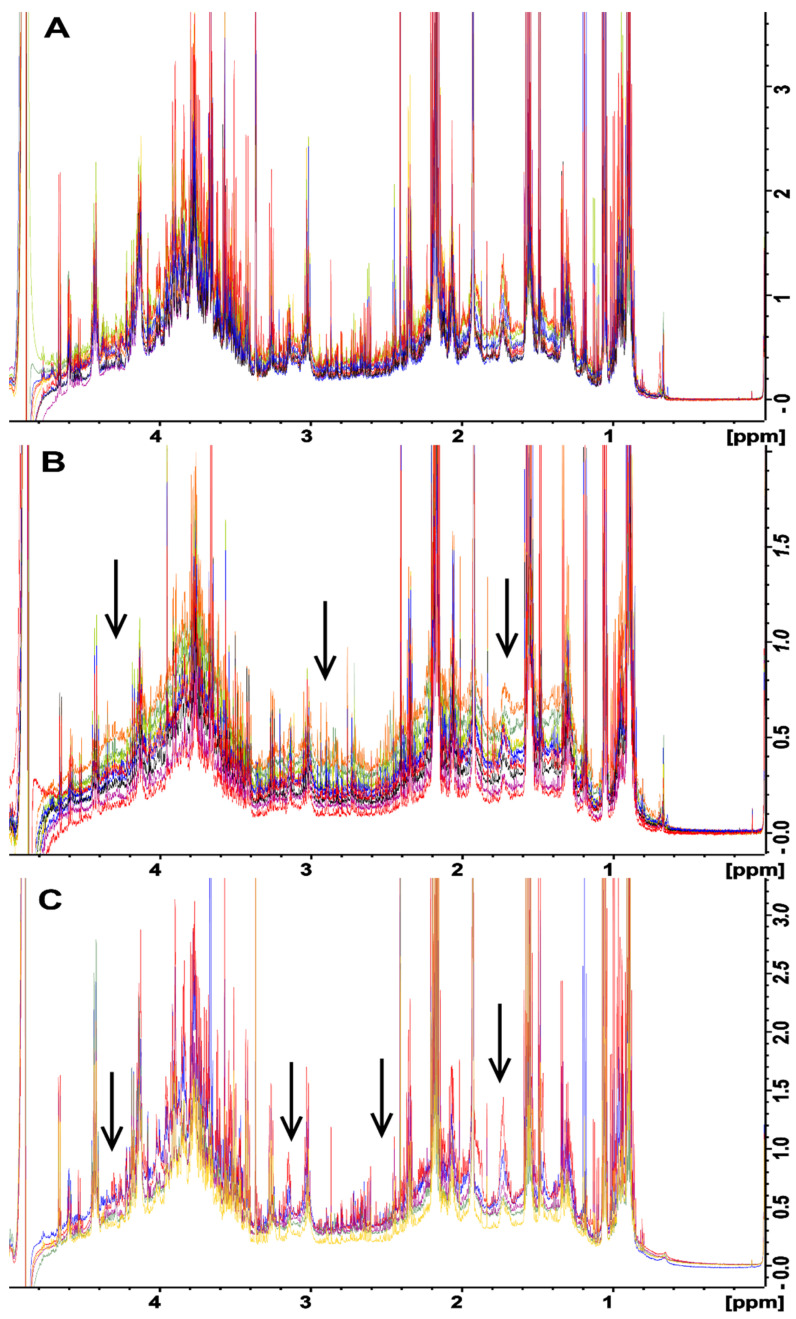
Overlaid NMR spectra in the 1–5 ppm range of rat feces samples that were each divided into three equivalent aliquots and were processed by (**A**) ultrafiltration, (**B**) Bligh–Dyer extraction, and (**C**) no extraction. The fanning of the spectra baseline indicates variability between the methods, as the metabolome differences for each complete set of samples are identical. The vertical scale has been increased to better illustrate spectral fanning. Arrows point to areas of drastic spectral fanning.

All of the fecal samples that were extracted using the UF method showed a consistent baseline (Figure 2A) suggesting that all macromolecules were removed from the samples. However, upon close examination, the samples that were processed using both the BD (Figure 2B) and no extraction (Figure 2C) methods displayed a baseline distortion that was most prominent in the 1–5 ppm region of the spectra, with this effect being most pronounced for the BD method (Figure 2B). The BD and no extraction spectra were produced from the same samples that were used for testing the UF method, for which a consistent baseline was observed, suggested that the observed variability was due to the extraction method and was most likely caused by residual macromolecules in the samples. For example, lipids in the sample gave broad signals mainly at 0.8–0.9 ppm, 1.1–1.4 ppm, and 5.2–5.4 ppm from CH_3_ groups, (CH_2_)_n_ groups, and = CH groups, respectively [26]. Thus, even though the no extraction method provided more intense metabolite peaks as compared to the UF method (Figure 1), the quantification of these results will not be reliable due to baseline distortions, as broad signals cause the integral of the peaks to be increased, making quantification inaccurate [26]. It is possible that the observed distortions could have been caused by partial or complete saturation of the NMR signals from residual macromolecules. This would be caused by utilizing too short of a total relaxation delay between experiments. This would be an ideal cause of the problem, as the distortions could be easily removed by simply increasing the total relaxation delay between NMR scans. To test this, the NMR experiments were repeated using twice the relaxation delay, and this change did not remove or reduce the observed baseline distortions (data not shown). This indicates that partial or complete saturation of the NMR signals from residual macromolecules was not the cause of this problem; rather, it is most likely the presence of varying amounts of residual macromolecules across samples due to the extraction process was responsible for these distortions. It is noteworthy that the macromolecular content was not measured for each of the different extraction methods. Future studies should measure macromolecule content utilizing alternative techniques such as a Bradford assay for proteins and gas or liquid chromatography combined with mass spectroscopy for lipids.

To investigate the effects of inter-species variability on metabolite extraction from fecal samples, the same test was conducted with fecal samples obtained from chickens (Supplemental Figure S1). A trend similar to that obtained for samples from rats was observed. That is, the baseline of the UF spectra showed few to no distortions, while the BD and no extraction methods exhibited significant baseline distortions in the 1–5 ppm range of the spectra. This indicated that the problem is not species-specific, and it needs to be considered when processing fecal samples regardless of the species under examination.

### 2.3. In Silico Baseline Correction Removed the Baseline Distortions from Macromolecules

**Figure 3:**
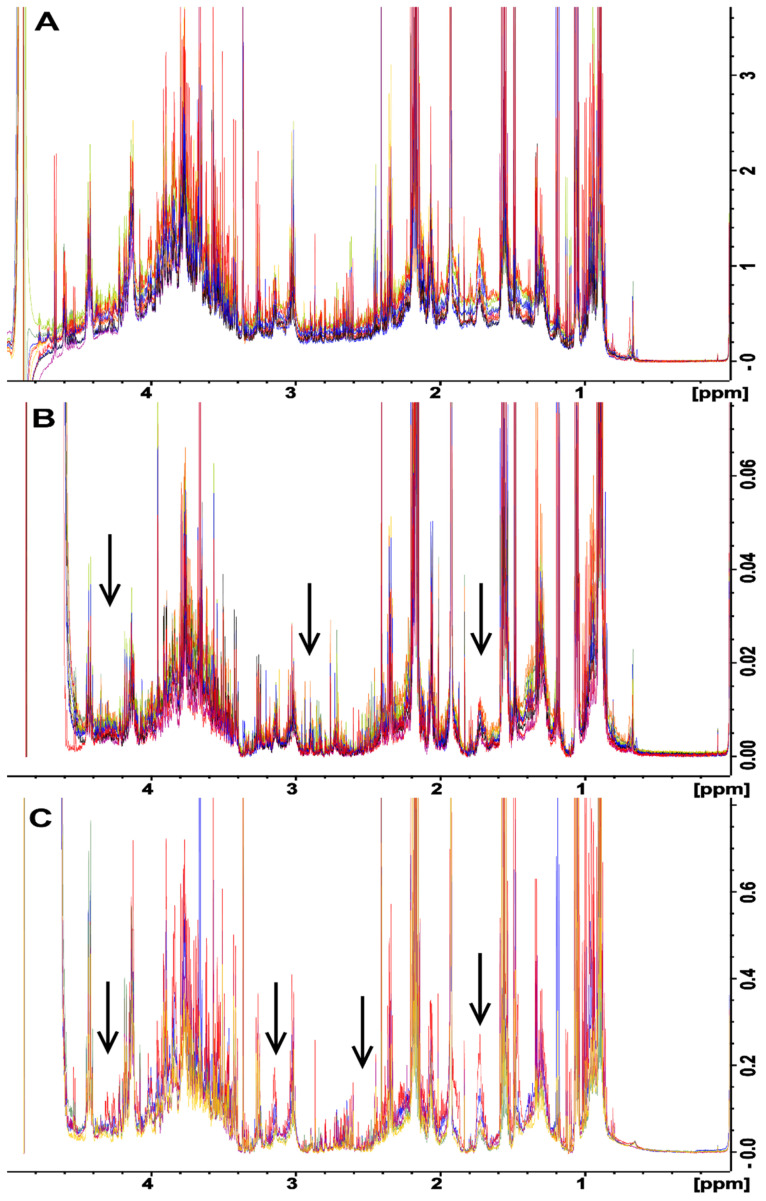
Comparison of NMR water-soluble metabolite spectra in the 1-5 ppm of range of rat feces that were divided into three equivalent aliquots and were processed by (**A**) ultrafiltration, (**B**) Bligh–Dyer extraction, and (**C**) no extraction. Spectra in (**B**,**C**) were processed after data collection using in silico baseline correction with a filter width of 175 Hz. The baseline correction has removed or greatly reduced the distortions observed for the same samples prior to correction. All three sample processing methods now produce a consistent baseline across all samples. Arrows correspond to those in Figure 2 showing areas where spectral fanning has been reduced or eliminated by in silico baseline correction.

A relatively unexplored option for removing the baseline distortions observed in the spectra obtained for the BD or no extraction methods is the application of in silico baseline correction. Baseline correction is an attractive alternative to the molecular cutoff filters used in the UF method, as these filters can be quite expensive on a per-sample basis as compared to baseline correction, which has no per-sample cost. However, the correct filter width must be selected to achieve optimal baseline correction (i.e., that most closely resembles the baseline obtained with the UF method). Several different filter widths for in silico baseline correction were tested, and a filter width of 175 Hz was observed to produce a spectral baseline that was most similar to the UF spectrum (Supplementary Figure S2). Subsequently, baseline correction was applied using the 175 Hz filter width to each of the spectra obtained for rat fecal samples processed with the BD and no extraction methods (Figure 3). Both the BD and no extraction methods followed by in silico baseline correction (Figure 3B,C, respectively) produced baselines with few to no distortions across samples, and both closely resembled the spectrum acquired for the UF method (Figure 3A). It is noteworthy that the application of baseline correction appeared to cause two additional changes. Firstly, the application of baseline correction resulted in a larger section of the spectrum around the water peak being unusable, which is a result of the way in which the filter is applied (see Section 3.7). Secondly, the overall height of the baseline between 1 and 5 ppm was slightly reduced compared to that of the UF method. The latter of these two could be seen as a benefit with respect to consistent metabolite quantification.

In silico baseline correction with a filter width of 175 Hz was also applied to the chicken fecal samples prepared using the BD and no extraction methods (Supplemental Figure S3B,C, respectively). In the case of chicken feces, when combined with in silico baseline correction, both the BD and no extraction methods provided more consistent spectral baselines than BD and no extraction alone. However, the spectral baselines were not as consistent as the spectral baseline produced by UF. This indicates that in silico baseline correction can be effectively applied to correct baseline distortions regardless of the species under investigation, but the choice of filter width is most likely species specific.

Another method that is frequently used to accommodate samples with large quantities of macromolecules, such as whole blood samples, is the Carr Purcell Meiboom Gill (CPMG) pulse sequence for data acquisition. This pulse sequence functions through the application of multiple repeated Hahn echoes, whereby the NMR signals from macromolecules, which have much more rapid transverse relaxation times (T_2_), are fully relaxed during the echo evolution period, whereas small molecule metabolites are not (i.e., they have much longer T_2_ times) [43]. This is known as relaxation editing or filtering, and the resulting spectra do not contain the signals from the macromolecules. However, there are some issues with the application of the CPMG pulse sequence. Firstly, if the sample has high protein content, the proteins can bind to small molecules, and this subsequently reduces the T_2_ times and also broadens the small molecule metabolite NMR peaks, leading to an overestimation of metabolite quantities [44,45]. Secondly, the application of the pulse sequence-based relaxation method inherently removes the ability to accurately quantify absolute concentrations of metabolites, as their signals are differentially reduced by the application of the Hahn echo train (i.e., each metabolite is differentially affected by this relaxation editing) [46,47,48]. As a result, the application of the CPMG pulse sequence leads to data that can only be quantified on a relative scale. For example, only the metabolite concentration normalized to the total metabolome can be reported. For these reasons, our findings show that both the no extraction and BD method followed by in silico baseline correction offer a cost-effective choice as an alternative to the UF method.

### 2.4. Different Extraction Methods Produce Different Fecal Metabolomes

**Figure 4:**
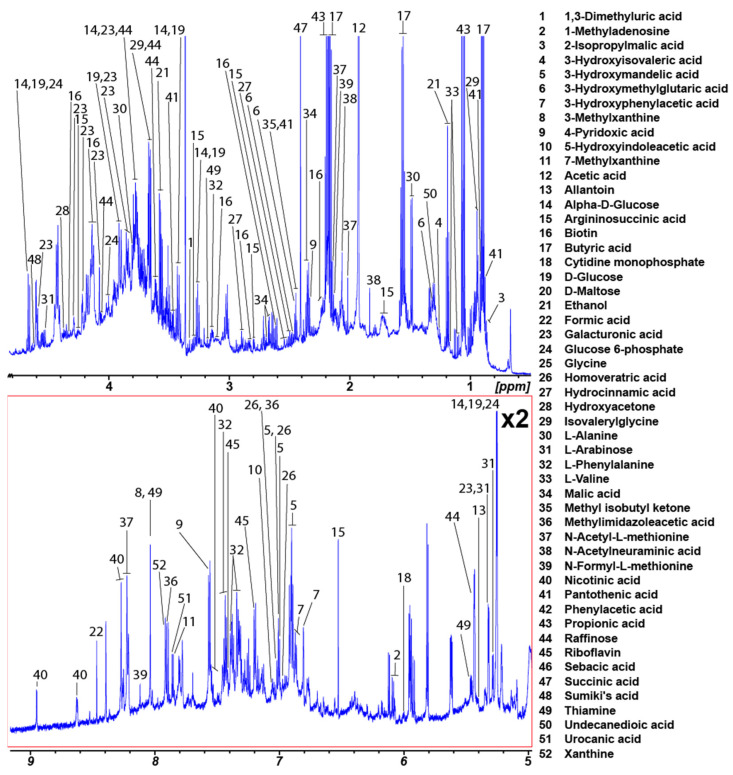
Average water-soluble rat fecal metabolome. The numbers in the figure correspond to Supplemental Table S2. Only metabolites that were present in spectra for the five methods evaluated and had a percentage occurrence of 50 or higher are included.

In total, 346 unique metabolites were identified from the rat fecal spectra (Supplementary Table S1). A total of 130 of these metabolites were common to the five extraction methods, and only 52 had an occurrence of 50 percent or higher. The average rat fecal metabolome of these 52 metabolites is shown in Figure 4 with concentration details for each extraction method provided in Supplemental Table S2. Thirty-four metabolites were identified in the chicken fecal spectra from the custom library of 37 metabolites (Supplemental Figure S4, Supplemental Table S3).

The concentrations observed for the no extraction and UF methods were similar for most metabolites and higher relative to the other three methods. However, in some cases, metabolite concentrations were higher for the no extraction method as compared to UF. In the case of chickens, the concentrations of metabolites obtained using the no extraction method were consistently higher than those obtained using both the UF and BD methods (Supplemental Table S3). The higher concentrations observed for the no extraction method could be caused by the incomplete removal of fats, which makes the peak areas appear larger than they are. Alternatively, the lower concentrations observed for the UF extraction method could be caused by incomplete filtration, whereby the proteins that concentrate on the membrane bind to metabolites and prevent them from passing through the filter. This issue is exacerbated by the hydrophobic nature of the membranes, as the membranes adsorb proteins both on the surface and inside the pores due to membrane–protein interactions resulting from higher retention [49]. In addition, as filters are used, they accumulate the impermeable macromolecules that have been filtered out on top of the filter, and this can cause both concentration polarization and membrane fouling, which prevents the metabolites from passing through the filter [49]. This may be a more significant issue in samples that contain higher concentrations of macromolecules to be removed. For example, this might be the case for fecal samples obtained from animals that are being fed a protein-enriched diet. Lastly, the concentrations of metabolites from the BD methanol chloroform extraction were consistently lower than concentrations of metabolites from the UF and no extraction methods, likely due to the metabolites of interest being removed during the extraction. For example, the solvents used can result in the loss of some metabolites. In this regard, liquid–liquid extractions depend on the variable solubility of the metabolites in two immiscible solvents; however, there is no guarantee that the metabolites may not be more soluble in the chloroform than in the water, meaning that they may be lost when the chloroform evaporates. This is especially true for metabolites that may be bound to macromolecules. In addition, volatile metabolites can be lost during the drying step. Conversely, the no extraction method does not remove any of the interfering macromolecules. Metabolites can bind to these macromolecules, and as a result, their NMR signal is broadened or completely lost due to relaxation effects. It is also possible that any proteins in the samples will partially bind to the chemical shift reference TSP and lead to a splitting of the chemical shift indicator peak [50].

In the case of the baseline correction, the concentrations obtained via the no extraction and BD methods generally decreased, which supports the possibility that incomplete removal of macromolecules increased the calculated concentrations. However, there are a few key exceptions to this that were observed. In this regard, the concentration of galactose was increased for both methods followed by baseline correction, while glucose only exhibited an increase for the BD method. Similarly, the concentration of betaine increased only for the no extraction method with baseline correction. Lastly, following the application of baseline correction, a limited number of metabolites either became quantifiable or not. For example, methylamine, citrate, and pyruvate were only quantified using BD following the application of baseline correction, although they were quantified at levels that were near the detection threshold of NMR. Lastly, 2-hydroxybutyrate, which was not quantifiable for the no extraction method, was detectable following the application of baseline correction. These changes should be considered when choosing the extraction method for a targeted characterization of the fecal metabolome using ^1^H-NMR.

**Figure 5:**
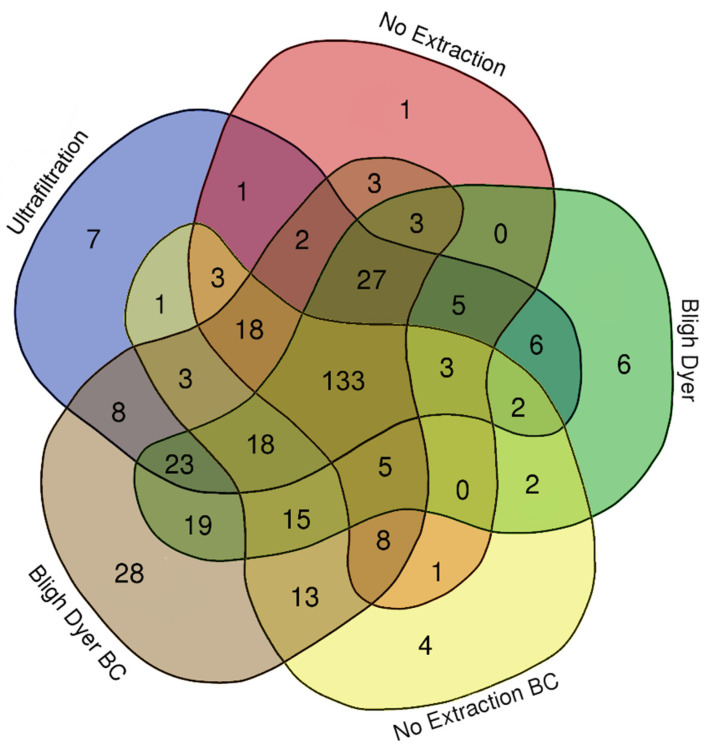
Venn diagram showing the number of water-soluble metabolites identified in rat feces that were processed by ultrafiltration, no extraction, Bligh–Dyer (BD) extraction, no extraction with baseline correction (BC), and BD with BC.

In summary, a diversity of metabolites were quantified in rat fecal samples by each method (Figure 5). The following numbers of metabolites were identified by each method in descending order: (i) BD method followed by baseline correction (313); (ii) the BD method with no baseline correction (267); (iii) the UF method (260); (iv) no extraction followed by baseline correction (229); and (v) no extraction (213). There were 46 metabolites that were unique to a single extraction method, with the largest number, 28, unique to the BD method when followed by baseline correction. Furthermore, more metabolites were identified for both the BD and no extraction methods when they were followed by in silico baseline correction in comparison to the same method without baseline correction (313 and 229 versus 267 and 213, respectively). These findings further support the use of either the UF method or the BD method followed by in silico baseline correction for NMR-based metabolomic analysis of feces, as they provide more metabolite information than the no extraction methods, and both have the potential to be applied to the study samples obtained from animals infected with pathogens.

### 2.5. Multivariate Modeling of the Different Extraction Methods

**Figure 6:**
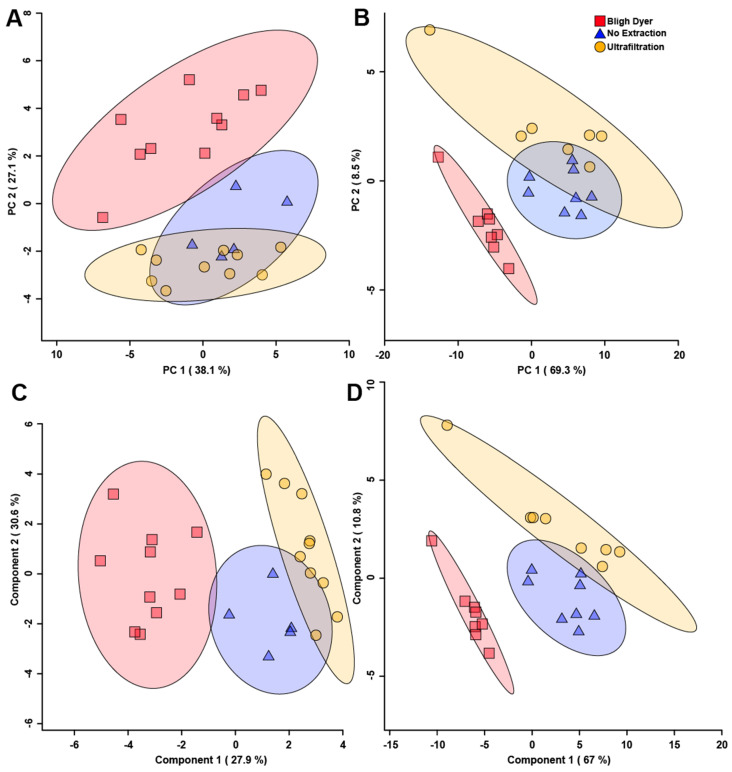
Principal component analysis (PCA) and partial least squares discriminant analysis (PLS-DA) score plots of all water-soluble fecal metabolite bins for rats (**A**,**C**); (*n* = 10) and chickens (**B**,**D**); (*n* = 8). The PCAs show unsupervised separation (**A**,**B**) and the PLS-DAs show supervised separation (**C**,**D**) among the three methods evaluated. For rats, *p* < 0.05, and for chickens, *p* < 0.001. Each symbol represents one fecal sample. For the PCAs, the data points are plotted with respect to the variability in the metabolome as given by the principal components. The x- and y-axes show principal components one and two, respectively, with brackets indicating percentage variance explained by each component. For the PLS-DAs, the data points are plotted using all bins, and the X and Y axes show the principal components with brackets indicating the percentage variance. The shaded ellipses represent the 95% confidence intervals for the score plots.

Principal component analysis (PCA) of the different extraction methods that were evaluated showed unsupervised separation between metabolite bins among the UF, BD, and no extraction methods for both rats (Figure 6A) and chickens (Figure 6B). This indicates that the extraction method chosen alters the metabolome obtained, and is concordant with the variation observed in the quantification of metabolites reported in both Supplemental Tables S1 and S3. For feces obtained from both species, the variations observed in the metabolite spectra obtained for the no extraction method more closely resembled the spectra obtained for the UF method. The spectra generated from the BD method showed a higher degree of separation relative to the no extraction and UF methods, as evidenced by minimal to no overlap in the 95% confidence intervals. The metabolites that contributed the most to the separation for rat fecal samples were butyric acid, formic acid, galacturonic acid, 1-methyladenosine, L-arabinose, glucose 6-phosphate, and alpha-D-glucose as determined from the PCA loadings plot (Supplemental Figure S5A). The metabolites that contributed the most to the separation for chicken fecal samples were 2-hydroxybutyrate, N-acetylglucosamine, acetate, and tyrosine (Supplemental Figure S5B).

**Figure 7:**
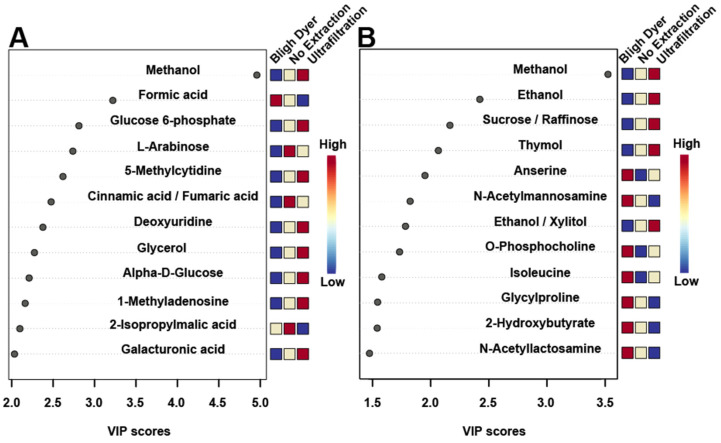
Variable importance to the projection (VIP) plots using all variables for rats (**A**) and chickens (**B**). The VIP plots show the top 12 fecal metabolites that contributed most to the separation seen in Figure 6C,D. The colored boxes along the right indicate whether the metabolite was up- or down regulated compared to the other extraction methods.

A supervised partial least squares discriminant analysis (PLS-DA) of the different extraction methods was carried out to better determine differences among the three extraction methods. The PLS-DA score plots show supervised separation among all three methods for both rats (Figure 6C) and chickens (Figure 6D). Variable importance to the projection (VIP) values obtained from the loading coefficients of the PLS-DA model indicated the top 12 metabolites that contributed to the observed group separation (Figure 7). In both rats (Figure 7A) and chickens (Figure 7B), the metabolite that contributed the most to supervised separation in Figure 6C,D was methanol. It makes sense that endogenous methanol might be lost during BD processing, as the solvent is left to evaporate, and for both the no extraction and UF methods, endogenous methanol would remain. There were no other metabolites in common between the two species, which is expected as rat feces is quite different from chicken feces, mainly in that rats eliminate nitrogenous waste via urine, and for chickens, nitrogenous waste is eliminated with the feces [51]. When discussing metabolomic differences observed in a study of feces, and in comparison to previous findings that utilized a differing extraction method, the metabolites presented in Figure 7 should be carefully considered.

## 3. Materials and Methods

### 3.1. Ethics Approval

The rat component of the study was reviewed and approved by the University of Lethbridge Animal Care Committee (Animal Use Protocol Review #1715). The chicken component of the study was reviewed and approved by the Agriculture and Agri-Food Canada (AAFC) Lethbridge Research and Development Centre (LeRDC) Animal Care Committee (Animal Use Protocol Review #1526 and #1903). The study was carried out in strict accordance with the recommendations established in the Canadian Council on Animal Care Guidelines.

### 3.2. Animal Protocol

Long–Evans rats were born and housed in the University of Lethbridge vivarium on 12:12 h day/night cycle in standard polycarbonate cages on corn chip bedding. The ambient temperature in the animal room was 22 °C, and animals had access to food and water ad libitum. White Leghorn chickens were born and housed in the LeRDC vivarium on 16:8 hr day/night cycle. Chickens were maintained in individually ventilated cages (Techniplast, Montreal, QC, Canada) on autoclaved wood shavings (United Farmers of Alberta Co-operative Ltd., Lethbridge, AB, Canada). The ambient temperature in the animal room was 30 °C for the first 2 days, 28 °C for the next 2 days, and 26 °C thereafter, and birds had access to food (Hi-Pro Feeds, Lethbridge, AB, Canada) and water ad libitum.

### 3.3. Sample Preparation—Comparing Methods without Variation across Individuals

Recently excreted fecal pellets were collected from a single adult Long–Evans rat at a single time point, and placed at −80 °C within 20 min of collection. Frozen samples were later divided into four equal aliquots with the goal of conducting analyses on samples with identical metabolomes. The four fecal subsamples were thawed on ice, mixed with metabolomics buffer at a 2:1 volume to mass ratio, vortexed until the sample became homogenous, centrifuged at 14,000× *g* for 20 min, and the supernatant was removed and hereafter is referred to as “fecal water”. The metabolomics buffer consisted of a 4:1 ratio of mono and dibasic potassium phosphate salts (K_2_HPO_4_/KH_2_PO_4_) in H_2_O with 3 mM sodium azide (NaN_3_). The resulting pH of this buffer was 7.4; sodium azide was added as an anti-microbial agent. Fecal water samples were then stored at −80 °C until processed. Water soluble metabolites were isolated from the fecal water using the following four techniques: (1) no extraction (2) UF, (3) BD extraction, and (4) fatty acid favoring extraction. In the case of the no extraction method, 275 μL of each fecal water sample was used to prepare the NMR sample without further processing.

#### 3.3.1. Ultrafiltration

Amicon Ultra 0.5 mL Centrifugal Filters (Merck Millipore, Cork, Ireland) with a molecular weight cutoff of 3 kDa were utilized. Each filter was washed by adding 500 μL of Millipore water (Merck Millipore) to the filter, and centrifuging at 14,000× *g* for 5 min. This washing step was repeated 10 times in order to ensure that all the glycerol in the filter had been removed [36,52]. Following the wash step, 350 μL of the fecal water was added to the filter and centrifuged at 14,000× *g* for 30 min at 4 °C. A 275 μL aliquot of the fecal water filtrate was set aside for NMR sample preparation.

#### 3.3.2. Bligh–Dyer Methanol-Chloroform Extraction

In a 2 mL tube, 275 µL of each fecal water sample was combined with 387.5 µL of methanol and 343.8 µL of chloroform. The tube was then vortexed and stored at −20 °C for 15 min to precipitate proteins, and then centrifuged at 15,300× *g* for 15 min at 4 °C. The supernatant from each sample was decanted into a new tube containing equal volumes of 343.8 µL of chloroform and deionized water. This mixture was then vortexed briefly and centrifuged at 6700× *g* for 5 min at 4 °C. Following centrifugation, 1 mL of the top aqueous layer containing the water-soluble metabolites of each sample was pipetted into a new tube, and samples were placed in a nitrogen gas flow box for 5–6 days to dry. Once dry, samples were rehydrated in 275 μL of deionized water, and the rehydrated samples were set aside for NMR sample preparation.

#### 3.3.3. Fatty Acid Removal Favoring Liquid–Liquid Extraction

In a 2 mL tube, 275 μL of each fecal water sample was combined with 1500 μL of methanol and 1500 μL of chloroform. The sample tube was vortexed and stored at −20 °C for 15 min to allow precipitation of proteins and then centrifuged at 15,000× *g* for 15 min at 4 °C. The supernatant from each sample was then transferred to a new microcentrifuge tube containing 375 μL of methanol, 375 μL of chloroform, and 475 μL of deionized water. Each sample was vortexed and centrifuged at 6800× *g* for 5 min at 4 °C. After centrifugation, 1 mL of the top layer containing the water-soluble metabolites of each sample was pipetted into a new tube, left to dry in a nitrogen gas flow box, and rehydrated as described above.

### 3.4. Sample Preparation—Replication of Preparation Methods across Multiple Samples

Individual fecal samples were obtained from rats (*n* = 10) and chickens (*n* = 8). Each fecal sample was split into three aliquots (i.e., subsamples) with each having a mass of at least 150 mg, and each aliquot was used to prepare fecal water as described previously. The following three methods were evaluated for variation within and across fecal samples by species: (1) no extraction; (2) UF; and (3) BD extraction as described previously. In silico baseline correction was applied to the spectra obtained from fecal water samples processed by the BD and no extraction methods. This allowed for a comparison of the application of the in silico baseline correction filter to the spectra obtained using the UF method.

### 3.5. Sample Preparation for ^1^H-NMR Spectroscopy

From each sample, 275 μL was mixed with 120 μL of deuterium oxide (D_2_O), containing 0.027% *w*/*v* sodium 3-trimethylsilylpropanoate-2,2,3,3-d_4_ (TSP), and 205 μL of metabolomics buffer (total volume of 600 μL, pH 7.4); TSP was used as a chemical shift reference for ^1^H-NMR spectroscopy. The solution was vortexed and then centrifuged at 12,000× *g* for 5 min at 4 °C to pellet particulate matter. Following centrifugation, a 550 μL aliquot of the supernatant was loaded into a 5 mm NMR tube.

### 3.6. NMR Data Acquisition and Processing

Spectra were obtained on a 700 MHz Bruker Avance III HD spectrometer using the 1-D NOESY gradient pulse pre-saturation water suppression pulse sequence ‘noesygppr1d’ with 10 msec mixing time. Each sample was run for 512 scans to a total acquisition size of 128 k, a spectral window of 20.5 ppm, a total data acquisition time of 4.56 sec, a transmitter offset of ≈4.7 ppm, and a recycle delay of 1 sec (total T1 relaxation recovery time of 5.56 sec). Both the transmitter offset and mixing time utilized for water suppression were optimized for fecal samples prepared using all three of the processing methods outlined above. In addition, the transmitter offset was optimized to ensure optimal water suppression prior to the start of data collection on each sample, hence the reported offset of ≈4.7 ppm. Prior to NMR data acquisition, three-dimensional and one-dimensional shimming experiments were conducted on the samples to correct for any inhomogeneities in the static magnetic field. To ensure a minimal spectral resolution, a test spectrum was collected on each sample immediately following shimming. This test spectrum was subjected to line width measurements on the TSP peak at 50%, 25%, and 10% of the max height, and had to meet a minimal line width of 1.0 Hz, 1.8 Hz, and 3.0 Hz, respectively. If this minimum specification was achieved, full data collection proceeded; if not, the shimming process was repeated until the specification was met. As a result of the stringency of the minimum specification, only five of the rat samples processed using the no extraction method produced useable spectra, most likely due to proteins in the samples binding to the TSP peak and causing broadening. All measurements were recorded using a Bruker triple resonance TBO-Z probe at 22 °C. The Bruker automation program “pulsecal” was used on each sample before data acquisition to guarantee that the 90-degree pulse was calibrated correctly, ensuring quantitative and comparable data across samples [53]. All spectra were initially processed inside Bruker TopSpin software (v. 3.5 pl 7). The spectra were zero filled to 256 k points, automatically phased using only zero-order phase correction, baseline corrected using a first-order polynomial, and line-broadened using an exponential decay function of 0.3 Hz [54]. Spectra were then exported to MATLAB (MathWorks, Natick, MA, USA) as ASCII files and underwent dynamic adaptive binning [55], followed by manual inspection and correction. Spectral binning resulted in 391 and 384 spectral bins for rat and chicken fecal samples, respectively. The dataset was normalized using the constant sum method, where each spectrum is set to have a unit total area, and each bin is a fraction of the total spectral integral (with the regions corresponding to water removed). The data set were then Pareto-scaled to reduce the influence of intense peaks while emphasizing weaker ones. All peaks were referenced to TSP (0.00 δ) [53]. Principal component analyses (PCAs) and partial least squares discriminant analyses (PLS-DAs) were performed on the complete set of fecal NMR spectral bins using MetaboAnalyst (v. 5.0) [56].

### 3.7. In Silico Baseline Correction

The baseline correction algorithm provided with the Bruker AssureNMR software (v. 2.2; Bruker, Billerica MA, USA), referenced as underground baseline correction by the manufacturer, was used to create a python macro that was applied in Bruker’s TopSpin software (v. 3.5 pl 7). The first step in this macro was to enter a filter width in hertz (Hz) applicable to the baseline correction. This width was then converted from Hz to the number of spectral data points by dividing the width by the digital resolution (number of spectral points per Hz). For each datum point in the spectrum, the algorithm determines a minimum intensity value (baseline) by searching to the left and right of the datum point by the number or points set as the filter width. This minimum value was then subtracted from the current datum point, and the process was repeated until all data points had been searched. Following this process, an average value was calculated from the first corrected point to that point plus 1/16th of the total spectral points. This average value was then subtracted from every datum point in the spectrum.

### 3.8. Metabolite Quantification

Metabolites were identified and quantified using Chenomx NMR Suite 8.5 standard (Edmonton, AB, Canada) with custom libraries of metabolites that were previously identified in mammalian [21] and chicken [57] feces. The mammalian library contained 415 metabolites previously detected in feces and present as NMR spectra in either the Human Fecal Metabolome Database (HFMDB) [58] or Chenomx database. The chicken library contained 37 metabolites previously detected in chicken feces [57] and present as NMR spectra in the Chenomx database. The concentration of each metabolite was calculated using the known internal concentration of the TSP peak for each sample (0.37 mM).

## 4. Conclusions

The aims of the current study were to: (i) examine the variance observed in the fecal metabolomic data obtained from NMR following the application of the most common small molecule water-soluble metabolite extraction methods; (ii) assess the utility of applying in silico baseline correction as a means to deal with incomplete removal of macromolecules from fecal samples; and (iii) provide a recommendation for the best extraction method, in terms of efficiency, reproducibility, and cost, to be utilized for NMR-based metabolomic studies of feces. Results obtained showed that the no extraction method provided the best signal-to-noise ratio as compared to either the UF or BD extraction methods. However, an examination of inter-sample variability showed that both the no extraction and BD methods resulted in baseline distortions that were caused by the incomplete removal of macromolecules during extraction. Notably, baseline distortions were not evident for samples extracted using the UF method. In addition, the baseline distortions observed for both the no extraction and BD methods were shown to be species-independent, as they were observed for fecal samples obtained from both rats and chickens. The application of in silico baseline correction with a filter width of 175 Hz to the spectra obtained following the no extraction and BD methods effectively removed the distortions and produced spectra with a consistent baseline similar to that obtained with the UF method, regardless of the species investigated. When determining the most practical extraction method, it is important to consider if the sample being extracted potentially contains pathogens. The UF method can be utilized in the case of both normal and pathogen-containing samples, whereas the no extraction method cannot be used on samples potentially containing pathogens, and the BD method must be tested on each pathogen to ensure that the solvents used effectively kill the pathogen. To this end, we found that the most versatile, reproducible, and efficient method to extract water-soluble small molecule metabolites from fecal samples was the UF method. However, salient disadvantages of the UF method were its higher cost and the potential for membrane fouling. Although the no extraction and BD methods were subject to significant baseline distortion, the application of in silico baseline correction largely negated the distortion. Thus, these two methods with baseline correction should be explored further, including their applicability to characterize the fecal metabolome of other species as well as non-fecal samples using ^1^H-NMR-based metabolomics.

The findings of the current study further support the need to carefully choose an extraction method and reinforce that any fecal metabolomic findings reported in the literature must take the extraction method used into consideration. The similarity between the spectra obtained using the no extraction and UF methods further supports the use of either UF, or the no extraction method with in silico baseline correction, to characterize the water-soluble metabolome in feces using ^1^H-NMR metabolomics.

## Data Availability

The data presented in this study are available on request from the corresponding authors, as it has not been uploaded to an online database.

## Supplementary Materials

The following supporting information can be downloaded at: www.mdpi.com/article/10.3390/metabo12020148/s1, Figure S1. Overlaid NMR spectra of water-soluble metabolites in the 1–5 ppm region of chicken fecal samples that were divided into three equivalent aliquots and processed by (A) ultrafiltration, (B) Bligh–Dyer extraction, and (C) no extraction. The fanning of the spectra baseline indicates variability between the methods, as the metabolome differences for each complete set of samples were identical. The spectra were split at the water peak, and the vertical scale has been increased to better illustrate spectral fanning. Figure S2. The effect of different in silico baseline correction filter widths on a representative rat fecal spectrum. A filter width of 175 Hz was chosen, as it most closely resembled the spectrum produced by ultrafiltration, and is shown in blue superimposed on top of the spectrum obtained using a filter width of 175 Hz. Figure S3 Comparison of NMR water-soluble metabolite spectra in the 1–5 ppm range of chicken fecal samples that were divided into three equivalent aliquots and processed by (A) ultrafiltration, (B) Bligh–Dyer extraction, and (C) no extraction. In the case of B and C, the spectra were processed after data collection using in silico baseline correction with a filter width of 175 Hz. The baseline correction removed or greatly reduced the distortions observed for the same samples prior to baseline correction. Figure S4. Average water-soluble chicken fecal metabolome. Metabolite concentrations are listed in Supplemental Table S3. Figure S5. Principal component analysis (A, B) and partial least squares discriminant analysis (C, D) loading plots for rat (A, C) and chicken (B, D) feces. Numbers refer to the bins with loading values greater than 0.1 or less than −0.1. Table S1. Average (av.) concentrations in millimoles (mM) of all metabolites identified in rat feces that were processed by ultrafiltration (UF), no extraction (No Extract.), Bligh–Dyer (BD) extraction, no extraction followed by baseline correction (No Extract, BC), and BD followed by baseline correction (BD BC). Metabolites were identified and quantified using Chenomx. The numbers presented in brackets correspond to the standard deviation (STD) and percentage occurrence (% occur.) of each metabolite, respectively. The multiplicity listed corresponds to the chemical shifts provided for each metabolite, respectively. Table S2. Average concentrations (mM) of select metabolites in rat feces that were processed by ultrafiltration, no extraction without baseline correction, Bligh–Dyer (BD) extraction without baseline correction, no extraction with baseline correction, and BD with baseline correction. Metabolites were identified and quantified using Chenomx, and only metabolites with a percentage occurrence of 50 or higher are shown. Metabolite numbers correspond to Figure 4, and the numbers presented in brackets correspond to the standard deviation and percentage occurrence of each metabolite, respectively. Table S3. Average concentrations (mM) of all metabolites identified in chicken feces that were processed by ultrafiltration (UF), no extraction without baseline correction, Bligh–Dyer (BD) extraction without baseline correction, no extraction with baseline correction (BC), and BD with BC. Metabolites were identified and quantified using Chenomx. Metabolite numbers correspond to Supplemental Figure S4, and the numbers presented in brackets correspond to the standard deviation and percentage occurrence of each metabolite, respectively.
