# Supplementary material for: Fecal 1H-NMR Metabolomics: A Comparison of Sample Preparation Methods for NMR and Novel in Silico Baseline Correction
Source: Metabolites. 2022 Feb 5;12(2):148. doi: 10.3390/metabo12020148 (PMC8875708; doi:10.3390/metabo12020148)
Supplement: Supplementary file 1 [file metabolites-12-00148-s001.zip › metabolites-1515348-supplementary.pdf]

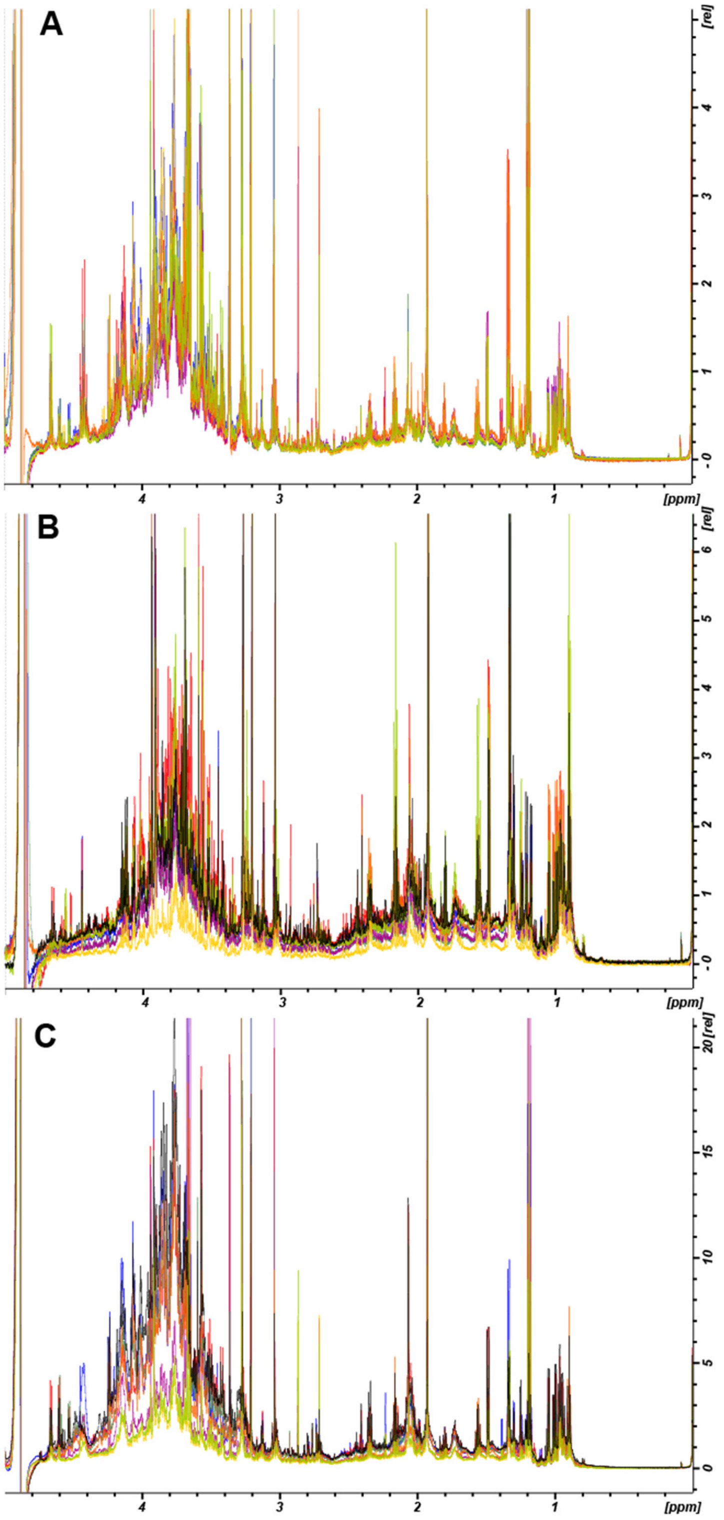

Figure S1. Overlaid NMR spectra of water-soluble metabolites in the 1-5 ppm region of chicken feces samples that were divided in three equivalent aliquots and were processed by (A) ultrafiltration, (B) Bligh-Dyer extraction, and (C) no extraction. The fanning of the spectra baseline indicates variability between the methods, as the metabolome differences for each complete set of samples are identical. The spectra have been split at the water peak, and the vertical scale has been increased to better illustrate spectral fanning.

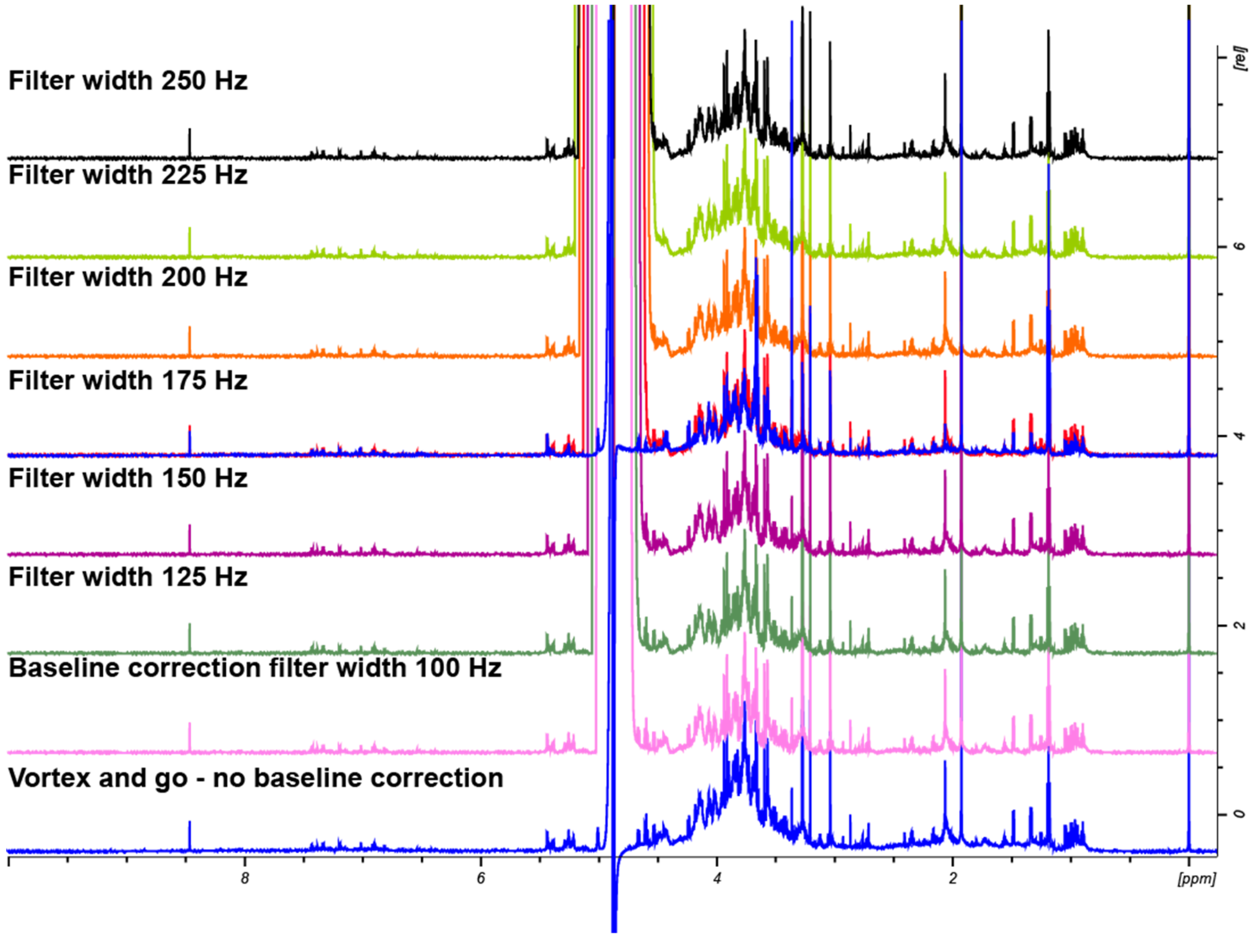

Figure S2. The effect of different *in silico* baseline correction filter widths on a representative rat fecal spectrum. A filter width of 175 Hz was chosen as it most closely resembled the spectrum produced by ultrafiltration and is shown in blue superimposed on top of the spectrum obtained using a filter width of 175 Hz.

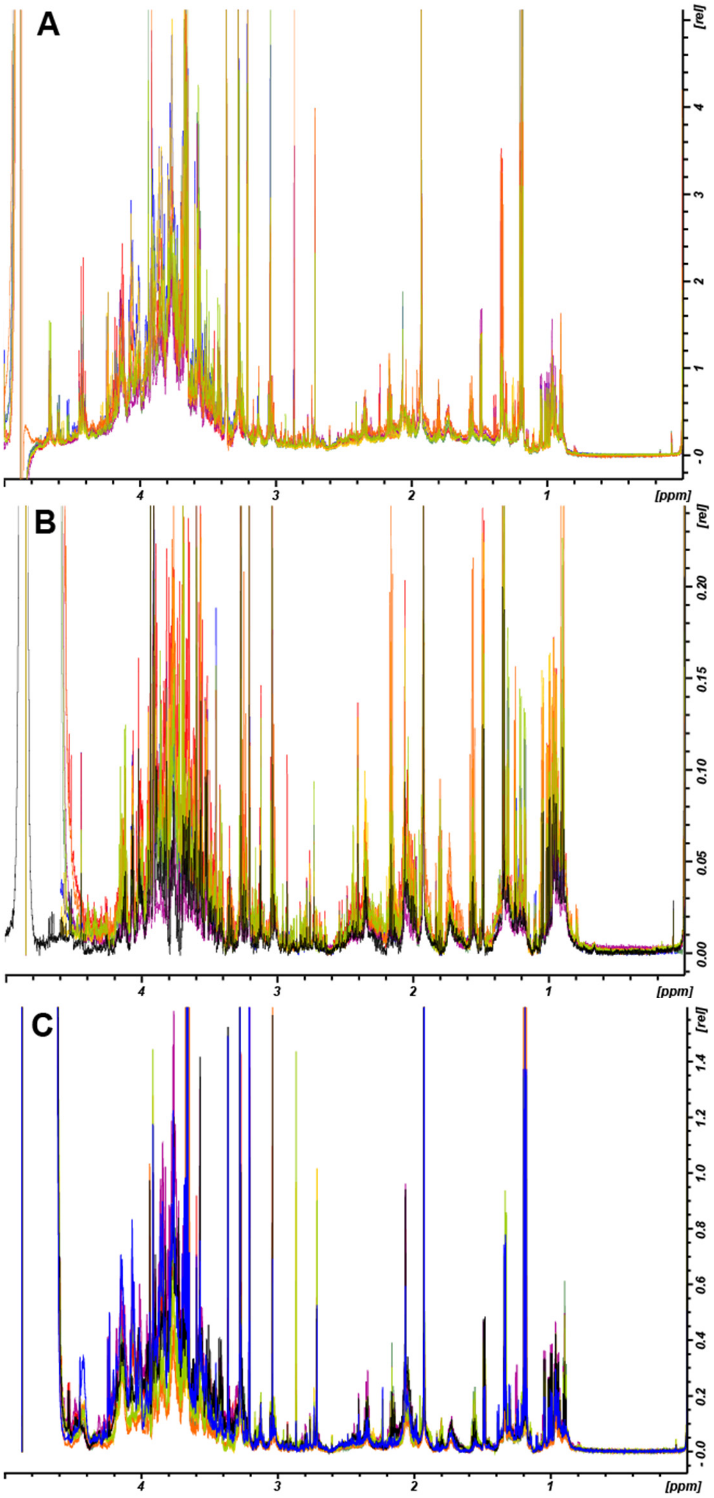

Figure S3. Comparison of NMR water-soluble metabolite spectra in the 1-5 ppm range of chicken feces samples that were divided into three equivalent aliquots and processed by (A) ultrafiltration, (B) Bligh-Dyer extraction, and (C) no extraction. In the case of B and C, the spectra were processed after data collection using *in silico* baseline correction with a filter width of 175 Hz. The baseline correction has removed or greatly reduced the distortions observed for the same samples prior to baseline correction.

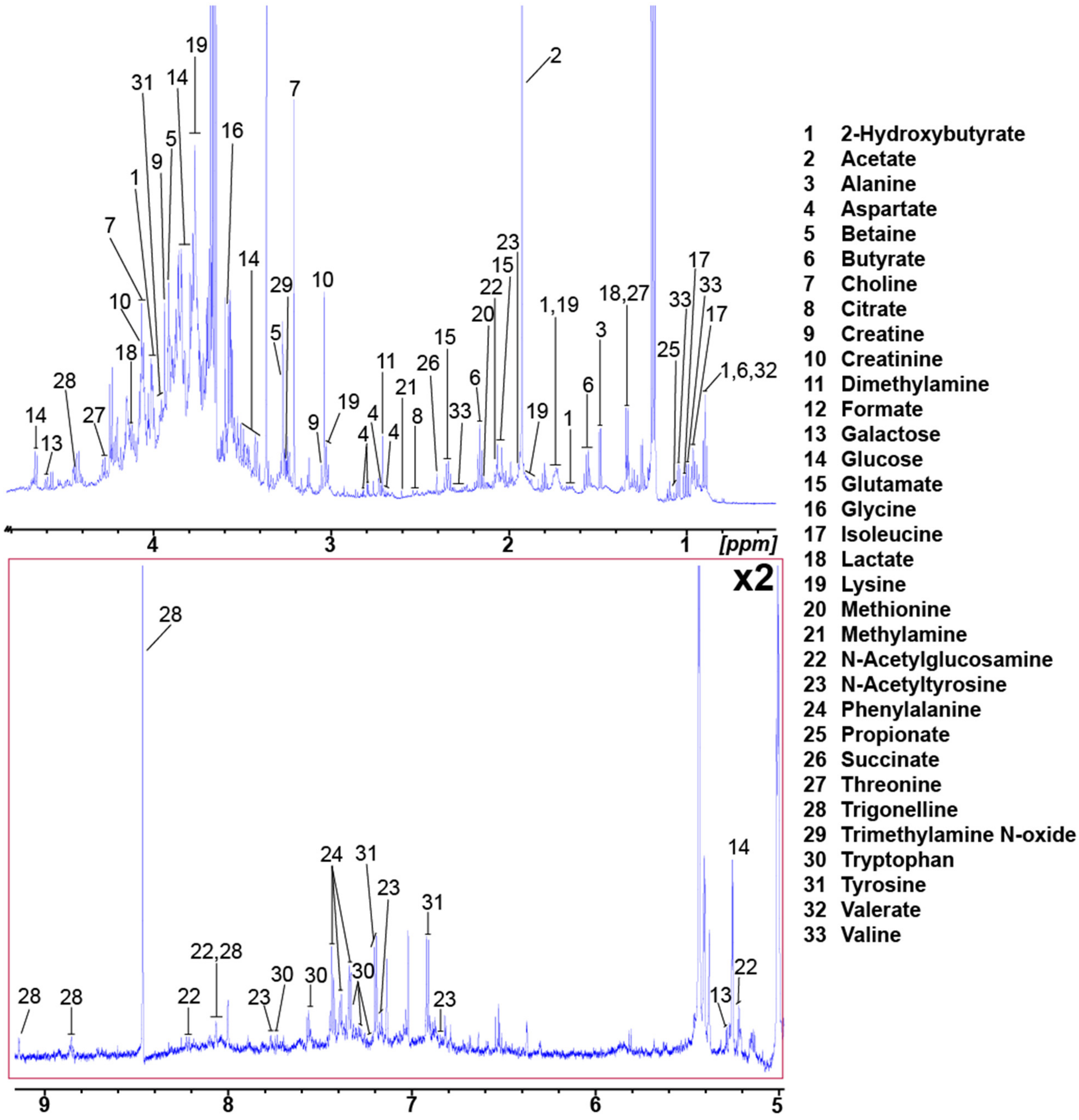

Figure S4. Average water-soluble chicken fecal metabolome. Metabolite concentrations are listed in Supplemental Table S3.

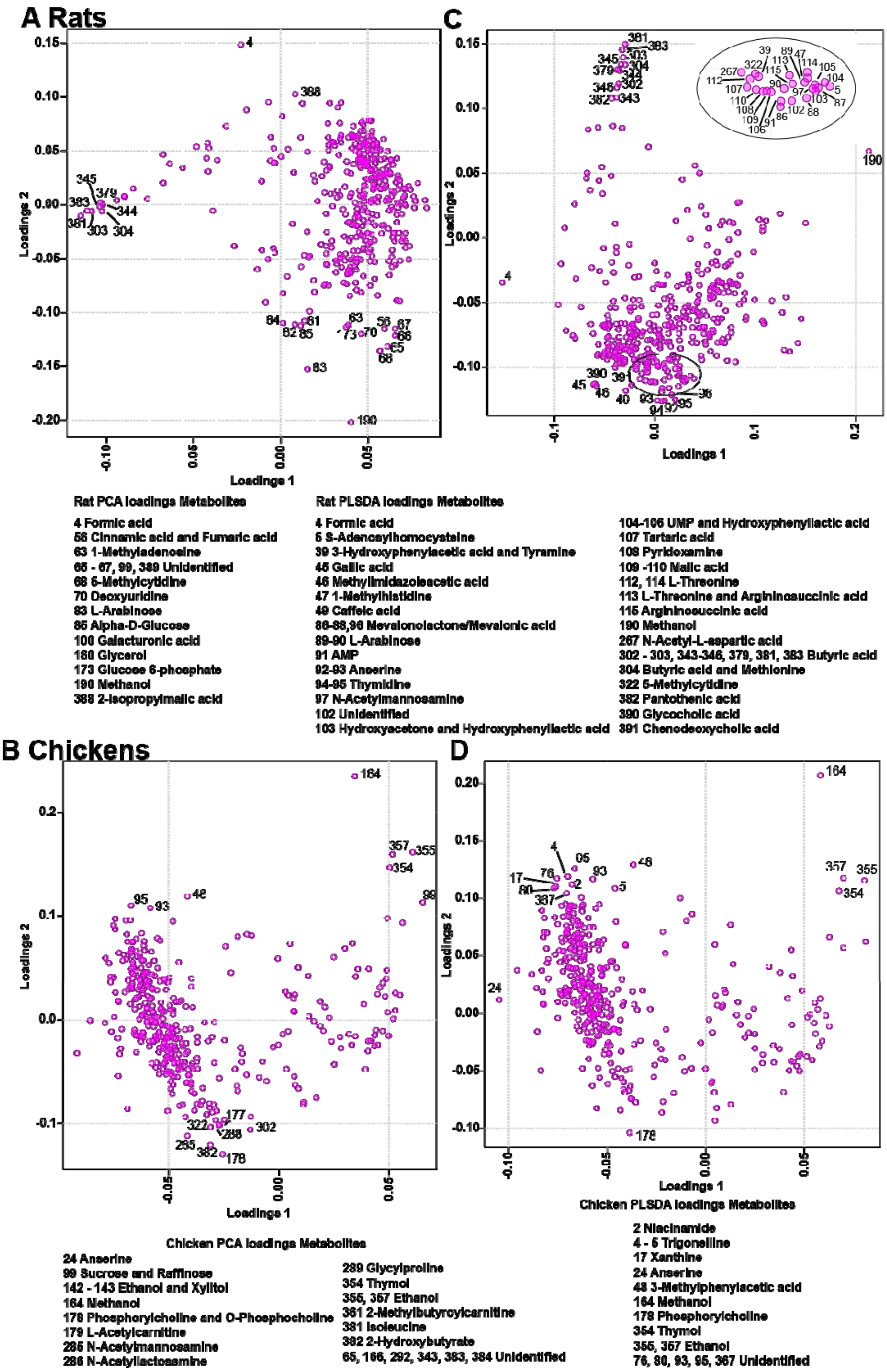

Figure S5. Principle component analysis (A, B) and partial least squares discriminate analysis (C, D) loadings plots for rats (A, C) and chickens (B, D). Numbers refer to the bins with loading values greater than 0.1 or less than -0.1.

Table S1. Average (av.) concentrations in millimolar (mM) of all metabolites identified in rat feces that were processed by ultrafiltration (UF), no extraction(No Extract.), Bligh-Dyer (BD) extraction, no extraction followed by baseline correction (No Extract. BC), and BD followed by baseline correction (BD BC). Metabolites were identified and quantified using Chenomx. The numbers presented in brackets correspond to the standard deviation (STD) and percent occurrence (% occur.) of each metabolite, respectively. The multiplicity listed corresponds to the chemical shifts provided for each metabolite, respectively.

Compound Name	Chemical Shift (ppm)	Multiplicity	No Extract. av mM (STD / % occur.)	BD av mM (STD / % occur.)	UF av mM (STD / % occur.)	No Extract. BC av mM (STD / % occur.)	BD BC av mM (STD / % occur.)
**Sebacic acid**	2.2, 1.5, 1.3	Triplet, Multiplet, Doublet	0.083 (0.038 / 100)	0.039 (0.011 / 90)	0.080 (0.033 / 100)	0.071 (0.034 / 80)	0.036 (0.014 / 80)
**Nervonic acid**	5.4, 2.4, 2.0, 1.6, 1.3, 1.3, 0.9	Triplet, Triplet, Quartet, Multiplet, Multiplet, Multiplet, Triplet	2.741 (0.885 / 100)	0.713 (0.025 / 20)	1.681 (0.391 / 70)	2.906 (0.558 / 100)	1.386 (0.565 / 90)
**5-Hydroxy-L-tryptophan**	7.4, 7.3, 7.1, 6.8, 4.0, 3.4, 3.4, 3.2	Doublet, Singlet, Doublet, Quartet, Quartet, Doublet, Doublet, Quartet	0.163 (0.088 / 60)	0.042 (0.018 / 80)	0.083 (0.034 / 100)	0.044 (0.027 / 40)	0.032 (0.008 / 70)
**Methyl isobutyl ketone**	2.4, 2.2, 2.1, 0.9	Doublet, Singlet, Multiplet, Doublet	1.837 (0.645 / 80)	0.445 (0.117 / 70)	1.171 (0.639 / 100)	0.926 (0.449 / 100)	0.350 (0.139 / 50)
**Erythrose**	5.3, 4.4, 4.2, 4.0, 3.8	Doublet, Doublet, Quartet, Triplet, Multiplet	5.015 (2.183 / 40)	0.539 (0.179 / 20)	2.611 (1.437 / 40)	2.772 (0.000 / 20)	1.051 (0.330 / 30)
**p-Cresol**	7.2, 6.8, 2.2	Doublet, Doublet, Singlet	0.086 (0.005 / 40)	0.015 (0.008 / 40)	0.054 (0.013 / 60)	0.041 (0.008 / 80)	0.012 (0.001 / 20)
**Urocanic acid**	7.9, 7.4, 7.3, 6.4	Singlet, Singlet, Doublet, Doublet	0.071 (0.034 / 100)	0.019 (0.005 / 70)	0.049 (0.020 / 90)	0.026 (0.013 / 60)	0.012 (0.003 / 60)
**m-Coumaric acid**	7.3, 7.2, 7.1, 6.9, 6.5, 6.5	Multiplet, Doublet, Triplet, Quartet, Singlet, Singlet	0.041 (0.014 / 40)	0.015 (0.003 / 70)	0.035 (0.009 / 60)	0.014 (0.002 / 40)	0.012 (0.004 / 80)
**Acetoin**	4.4, 2.2, 1.4	Doublet, Singlet, Doublet	0.169 (0.036 / 60)	0.040 (0.006 / 40)	0.183 (0.053 / 30)	0.158 (0.046 / 60)	0.042 (0.006 / 30)
**Acetylglycine**	8.0, 3.8, 2.0	Singlet, Doublet, Singlet	2.634 (0.642 / 100)	0.777 (0.276 / 100)	1.661 (0.542 / 100)	0.876 (0.517 / 40)	0.543 (0.173 / 90)
**Homovanillic acid**	6.9, 6.9, 6.8, 3.9, 3.4	Doublet, Doublet, Quartet, Singlet, Singlet	0.067 (0.038 / 60)	0.013 (0.006 / 80)	0.028 (0.021 / 40)	0.034 (0.011 / 80)	0.012 (0.004 / 100)
**Sucrose**	5.4, 4.2, 4.0, 3.8, 3.8, 3.7, 3.6, 3.5	Doublet, Doublet, Triplet, Multiplet, Triplet, Singlet, Quartet, Triplet	0.027 (0.032 / 60)	0.010 (0.005 / 30)	0.041 (0.014 / 90)	0.030 (0.037 / 80)	0.028 (0.013 / 90)
**Deoxycytidine**	7.8, 6.3, 5.9, 4.5, 4.0, 3.8, 3.7, 3.6, 2.4	Doublet, Triplet, Doublet, Multiplet, Multiplet, Quartet, Multiplet, Multiplet, Multiplet	0.016 (0.000 / 20)	0.012 (0.002 / 70)	0.018 (0.006 / 80)	0.015 (0.000 / 20)	0.013 (0.002 / 40)
**Valine**	3.6, 2.3, 1.0, 1.0	Doublet, Multiplet, Doublet, Doublet	0.101 (0.034 / 80)	0.033 (0.012 / 100)	0.138 (0.061 / 90)	0.066 (0.031 / 100)	0.031 (0.012 / 100)
**Leucine**	3.7, 1.7, 1.0	Multiplet, Multiplet, Triplet	0.249 (0.126 / 40)	0.062 (0.000 / 10)	0.096 (0.025 / 90)	0.123 (0.023 / 60)	0.041 (0.000 / 10)
**N-Acetyl-L-methionine**	8.2, 4.4, 2.6, 2.1, 2.0	Doublet, Multiplet, Multiplet, Singlet, Singlet	0.045 (0.019 / 100)	0.013 (0.006 / 70)	0.035 (0.006 / 60)	0.033 (0.005 / 100)	0.029 (0.058 / 100)
**N-Acetyl-L-aspartic acid**	7.9, 4.4, 2.7, 2.7, 2.5, 2.5, 2.5, 2.0	Doublet, Multiplet, Doublet, Doublet, Singlet, Doublet, Singlet, Singlet	0.230 (0.169 / 60)	0.119 (0.021 / 20)	0.179 (0.107 / 30)	0.120 (0.088 / 100)	0.202 (0.461 / 100)
**4,5-Dihydroorotic acid**	4.1, 3.0, 2.9, 2.8, 2.8	Triplet, Doublet, Doublet, Doublet, Doublet	0.028 (0.015 / 40)	0.062 (0.000 / 10)	0.154 (0.149 / 30)	0.059 (0.035 / 40)	0.022 (0.012 / 20)
**Thymine**	7.4, 1.9	Doublet, Doublet	0.050 (0.000 / 20)	0.028 (0.010 / 100)	0.075 (0.013 / 60)	0.071 (0.032 / 60)	0.019 (0.005 / 90)
**Phenylacetic acid**	7.4, 7.3, 3.5	Triplet, Triplet, Singlet	0.148 (0.051 / 60)	0.036 (0.012 / 80)	0.101 (0.019 / 80)	0.063 (0.014 / 60)	0.032 (0.008 / 90)
**Propionic acid**	2.2, 1.0	Quartet, Triplet	2.931 (0.807 / 100)	1.005 (0.177 / 100)	1.793 (0.436 / 100)	2.917 (0.776 / 100)	1.039 (0.159 / 90)
**1,3-Dimethyluric acid**	3.4, 3.3	Singlet, Singlet	0.045 (0.011 / 100)	0.009 (0.004 / 80)	0.022 (0.009 / 100)	0.007 (0.005 / 100)	0.002 (0.001 / 80)

**Undecanedioic acid**	2.2, 1.5, 1.3	Triplet, Triplet, Singlet	0.061 (0.031 / 100)	0.028 (0.009 / 100)	0.055 (0.023 / 100)	0.053 (0.036 / 100)	0.020 (0.014 / 80)
**Phenylalanine**	7.4, 7.4, 7.3, 4.0, 3.3, 3.1	Triplet, Multiplet, Doublet, Quartet, Quartet, Quartet	0.169 (0.116 / 100)	0.030 (0.009 / 70)	0.113 (0.046 / 100)	0.122 (0.056 / 100)	0.033 (0.008 / 70)
**p-Hydroxy-phenylacetic acid**	7.2, 6.9, 3.4	Doublet, Doublet, Singlet	0.071 (0.016 / 40)	0.014 (0.007 / 90)	0.035 (0.011 / 60)	0.024 (0.012 / 80)	0.020 (0.012 / 80)
**Acetic acid**	1.9	Singlet	13.393 (3.368 / 100)	4.602 (0.774 / 100)	8.367 (1.864 / 100)	13.386 (3.378 / 100)	4.652 (0.798 / 90)
**3,4-Dihy-droxybenzeneace-tic acid**	6.9, 6.8, 6.7, 3.4	Doublet, Doublet, Quartet, Singlet	0.025 (0.007 / 60)	0.034 (0.013 / 30)	0.088 (0.035 / 70)	0.039 (0.020 / 100)	0.033 (0.014 / 60)
**Ethanol**	3.6, 1.2	Quartet, Triplet	0.521 (0.445 / 60)	0.194 (0.100 / 90)	0.565 (0.306 / 100)	0.428 (0.415 / 80)	0.189 (0.094 / 90)
**Pyridoxal**	7.6, 6.6, 5.3, 5.2, 5.1, 2.5	Singlet, Doublet, Singlet, Singlet, Doublet, Singlet	0.022 (0.010 / 40)	0.003 (0.003 / 80)	0.015 (0.002 / 20)	0.002 (0.000 / 20)	0.002 (0.000 / 80)
**Histamine**	7.8, 7.1, 3.3, 3.0	Singlet, Singlet, Triplet, Triplet	0.052 (0.000 / 20)	0.006 (0.000 / 10)	0.022 (0.000 / 10)	0.015 (0.003 / 100)	0.011 (0.002 / 30)
**Indoxyl sulfate**	7.7, 7.5, 7.4, 7.3, 7.2	Doublet, Doublet, Singlet, Triplet, Triplet	1.226 (0.465 / 100)	0.400 (0.144 / 80)	0.676 (0.240 / 100)	0.261 (0.000 / 20)	0.231 (0.062 / 90)
**3-Cresotinic acid**	7.7, 7.3, 6.9, 2.2	Quartet, Doublet, Triplet, Singlet	0.204 (0.078 / 100)	0.113 (0.058 / 70)	0.108 (0.016 / 40)	0.116 (0.005 / 40)	0.093 (0.037 / 80)
**Glutamic acid**	3.7, 2.3, 2.1	Quartet, Multiplet, Multi-plet	0.521 (0.220 / 80)	0.169 (0.050 / 70)	0.347 (0.073 / 30)	0.307 (0.042 / 40)	0.098 (0.025 / 30)
**Uracil**	7.5, 5.8	Doublet, Doublet	0.226 (0.000 / 20)	0.248 (0.058 / 100)	0.470 (0.205 / 90)	0.212 (0.057 / 60)	0.243 (0.066 / 100)
**Methylimidaz-oleacetic acid**	7.9, 7.0, 3.7, 3.5	Singlet, Singlet, Singlet, Singlet	0.830 (0.411 / 100)	0.200 (0.109 / 100)	0.350 (0.180 / 100)	0.096 (0.047 / 100)	0.126 (0.070 / 90)
**Xylulose**	4.4, 4.2, 4.0, 3.6, 3.6, 3.6, 3.6	Quartet, Quartet, Doublet, Multiplet, Singlet, Singlet, Singlet	7.018 (5.220 / 100)	2.447 (0.778 / 20)	7.173 (4.898 / 100)	5.505 (3.016 / 100)	2.520 (1.140 / 60)
**Betaine**	3.9, 3.3	Singlet, Singlet	0.134 (0.066 / 40)	0.049 (0.015 / 20)	0.132 (0.049 / 60)	0.051 (0.015 / 100)	0.017 (0.009 / 80)
**Galacturonic acid**	5.3, 4.6, 4.4, 4.3, 4.2, 4.1, 3.9, 3.8, 3.7, 3.5	Doublet, Doublet, Doublet, Quartet, Quartet, Doublet, Quartet, Quartet, Quartet, Quartet	1.177 (0.284 / 100)	0.167 (0.057 / 100)	1.000 (0.251 / 100)	1.372 (0.286 / 100)	0.300 (0.043 / 90)
**Galactose**	5.3, 4.1, 4.0, 3.9, 3.8, 3.7, 3.6, 3.5	Doublet, Triplet, Doublet, Doublet, Quartet, Multi-plet, Quartet, Quartet	0.140 (0.037 / 40)	0.023 (0.016 / 50)	0.105 (0.014 / 60)	0.226 (0.061 / 100)	0.151 (0.000 / 10)
**Maltose**	5.4, 5.2, 3.9, 3.8, 3.7, 3.4, 3.4, 3.3	Doublet, Doublet, Multi-plet, Multiplet, Multiplet, Doublet, Singlet, Quartet	0.186 (0.147 / 60)	0.050 (0.035 / 50)	0.085 (0.061 / 80)	0.188 (0.171 / 40)	0.079 (0.028 / 70)
**Phenol**	7.3, 7.0	Triplet, Singlet	0.034 (0.000 / 20)	0.088 (0.030 / 90)	0.092 (0.016 / 30)	0.109 (0.050 / 100)	0.077 (0.020 / 100)
**5-Thymidylic acid**	7.8, 6.3, 4.2, 4.1, 2.4, 1.9	Doublet, Triplet, Multiplet, Multiplet, Multiplet, Dou-blet	0.083 (0.000 / 20)	0.020 (0.005 / 80)	0.067 (0.013 / 70)	0.033 (0.007 / 80)	0.021 (0.007 / 90)
**Allantoin**	6.0, 5.4	Singlet, Singlet	0.037 (0.015 / 80)	0.011 (0.004 / 60)	0.034 (0.011 / 100)	0.047 (0.012 / 100)	0.038 (0.013 / 60)
**Serotonin**	7.4, 7.3, 7.1, 6.9, 3.3, 3.1	Doublet, Singlet, Doublet, Quartet, Triplet, Triplet	0.026 (0.000 / 20)	0.031 (0.013 / 90)	0.054 (0.022 / 70)	0.015 (0.006 / 60)	0.018 (0.008 / 80)
**Aspartic acid**	3.9, 2.8, 2.8, 2.7	Quartet, Doublet, Doublet, Quartet	0.211 (0.094 / 80)	- (- / 100)	0.152 (0.081 / 100)	0.115 (0.034 / 100)	0.020 (0.011 / 80)
**N-Acetylneu-raminic acid**	4.0, 4.0, 4.0, 3.9, 3.9, 3.8, 3.8, 3.6, 3.5, 2.2, 2.1, 1.9, 1.8, 1.8, 1.8	Multiplet, Singlet, Singlet, Triplet, Doublet, Quartet, Multiplet, Quartet, Dou-blet, Quartet, Singlet, Sin-glet, Singlet, Singlet, Sin-glet	1.233 (0.268 / 100)	0.333 (0.106 / 100)	0.887 (0.210 / 100)	0.208 (0.188 / 100)	0.112 (0.059 / 100)
**FAD**	8.3, 7.9, 7.6, 7.6, 5.8, 4.5, 4.5, 4.3, 4.1,	Singlet, Singlet, Singlet, Singlet, Doublet, Triplet, Triplet, Multiplet, Singlet,	1.918 (0.959 / 100)	0.409 (0.214 / 90)	1.082 (0.514 / 90)	0.180 (0.000 / 20)	0.284 (0.057 / 90)

	4.1, 3.9, 2.4, 2.3	Multiplet, Quartet, Singlet, Singlet					
**Xylose**	5.2, 4.6, 3.9, 3.6, 3.5, 3.4, 3.3, 3.3, 3.3, 3.2	Doublet, Doublet, Quartet, Multiplet, Quartet, Triplet, Singlet, Singlet, Singlet, Quartet	0.121 (0.024 / 40)	0.036 (0.013 / 30)	0.081 (0.026 / 60)	0.082 (0.048 / 100)	0.018 (0.005 / 70)
**5-Hydroxyindole-acetic acid**	7.4, 7.2, 7.0, 6.8, 3.6	Doublet, Singlet, Doublet, Quartet, Singlet	0.094 (0.040 / 60)	0.015 (0.010 / 80)	0.046 (0.017 / 90)	0.014 (0.001 / 80)	0.010 (0.008 / 90)
**Thiamine**	9.5, 8.0, 5.5, 3.9, 3.2, 2.6, 2.5	Singlet, Singlet, Singlet, Triplet, Triplet, Singlet, Singlet	0.028 (0.017 / 60)	0.004 (0.003 / 70)	0.011 (0.005 / 70)	0.004 (0.002 / 60)	0.003 (0.001 / 60)
**Raffinose**	5.4, 4.2, 4.0, 4.0, 3.9, 3.9, 3.8, 3.8, 3.7, 3.7, 3.6, 3.5, 3.5	Doublet, Doublet, Multiplet, Doublet, Triplet, Multiplet, Multiplet, Multiplet, Singlet, Doublet, Quartet, Singlet, Singlet	0.634 (0.122 / 60)	0.121 (0.055 / 80)	0.239 (0.262 / 70)	0.378 (0.231 / 80)	0.285 (0.211 / 90)
**Histidine**	7.9, 7.1, 4.0, 3.3, 3.2, 3.2	Doublet, Singlet, Quartet, Doublet, Doublet, Quartet	0.111 (0.028 / 40)	0.027 (0.009 / 80)	0.077 (0.005 / 30)	0.013 (0.002 / 40)	0.016 (0.008 / 40)
**Malic acid**	4.3, 2.7, 2.6, 2.4, 2.4, 2.3	Quartet, Doublet, Doublet, Singlet, Doublet, Singlet	0.726 (0.233 / 100)	0.132 (0.078 / 100)	0.413 (0.166 / 100)	0.215 (0.160 / 100)	0.442 (1.226 / 100)
**Homoveratric acid**	7.0, 7.0, 6.9, 3.8, 3.5	Doublet, Doublet, Quartet, Doublet, Singlet	0.158 (0.051 / 100)	0.026 (0.014 / 50)	0.086 (0.013 / 80)	0.022 (0.001 / 40)	0.131 (0.313 / 100)
**7-Methylxanthine**	7.8, 3.9	Singlet, Singlet	0.747 (0.137 / 60)	0.236 (0.078 / 90)	1.155 (0.971 / 90)	0.760 (0.765 / 100)	0.227 (0.115 / 90)
**Glycerophospho-choline**	4.3, 3.9, 3.7, 3.6, 3.2	Multiplet, Multiplet, Multiplet, Quartet, Singlet	0.191 (0.000 / 20)	0.039 (0.000 / 10)	0.161 (0.046 / 60)	0.137 (0.070 / 100)	0.013 (0.005 / 70)
**Erythritol**	3.8, 3.7, 3.6	Quartet, Multiplet, Multiplet	0.554 (0.326 / 60)	0.221 (0.054 / 40)	0.583 (0.178 / 100)	0.381 (0.239 / 100)	0.151 (0.069 / 30)
**Butyric acid**	2.1, 1.5, 0.9	Triplet, Multiplet, Triplet	4.564 (2.255 / 100)	1.324 (0.467 / 100)	2.551 (1.217 / 100)	4.473 (2.367 / 100)	1.305 (0.457 / 90)
**4-Pyridoxic acid**	7.5, 4.5, 2.3	Singlet, Singlet, Doublet	0.033 (0.005 / 80)	0.007 (0.001 / 100)	0.022 (0.012 / 100)	0.024 (0.016 / 100)	0.007 (0.001 / 90)
**1-Methylhistidine**	7.7, 7.0, 4.0, 3.7, 3.2, 3.1, 3.1	Singlet, Singlet, Quartet, Singlet, Doublet, Doublet, Quartet	0.029 (0.000 / 20)	0.023 (0.014 / 100)	0.056 (0.017 / 100)	0.013 (0.002 / 80)	0.019 (0.007 / 100)
**Mevalonolactone**	4.6, 4.5, 2.7, 2.7, 2.7, 2.6, 2.1, 1.9, 1.4	Multiplet, Multiplet, Singlet, Singlet, Doublet, Doublet, Multiplet, Multiplet, Singlet	0.027 (0.000 / 20)	0.010 (0.002 / 50)	0.021 (0.004 / 60)	0.018 (0.006 / 100)	0.012 (0.016 / 100)
**Levoglucosan**	5.4, 4.6, 4.1, 3.7, 3.7, 3.5	Singlet, Doublet, Quartet, Quartet, Multiplet, Multiplet	0.118 (0.019 / 40)	0.018 (0.007 / 50)	0.092 (0.002 / 20)	0.157 (0.030 / 80)	0.074 (0.022 / 50)
**3-Hydroxymethylglutaric acid**	2.5, 2.4, 2.4, 2.4, 1.3	Singlet, Singlet, Singlet, Singlet, Singlet	0.184 (0.039 / 80)	0.068 (0.024 / 90)	0.094 (0.058 / 90)	0.128 (0.021 / 100)	0.035 (0.023 / 90)
**3-Methylxanthine**	8.0, 3.5	Singlet, Singlet	5.350 (1.078 / 80)	1.411 (0.457 / 80)	5.416 (0.907 / 70)	1.898 (0.972 / 100)	0.691 (0.397 / 80)
**3-Methylhistidine**	7.9, 7.0, 3.9, 3.7, 3.3, 3.3, 3.2	Singlet, Singlet, Quartet, Singlet, Doublet, Doublet, Quartet	0.140 (0.047 / 60)	0.025 (0.017 / 90)	0.061 (0.023 / 20)	0.011 (0.005 / 80)	0.013 (0.012 / 100)
**2-Heptanone**	2.5, 2.2, 1.5, 1.3, 0.9	Triplet, Singlet, Multiplet, Multiplet, Triplet	1.176 (0.243 / 60)	0.313 (0.092 / 80)	0.583 (0.255 / 60)	0.084 (0.000 / 20)	0.131 (0.026 / 30)
**Sumiki's acid**	6.9, 6.5, 4.6	Doublet, Doublet, Singlet	0.029 (0.020 / 80)	0.004 (0.002 / 80)	0.012 (0.013 / 80)	0.004 (0.001 / 40)	0.004 (0.002 / 50)
**Flavin Mononucleotide**	7.8, 7.6, 4.4, 4.3, 4.3, 4.1, 4.0, 3.9, 3.8, 2.5, 2.4	Singlet, Doublet, Multiplet, Multiplet, Quartet, Multiplet, Multiplet, Multiplet, Quartet, Singlet, Singlet	2.821 (0.000 / 20)	0.659 (0.335 / 90)	1.637 (0.411 / 60)	0.340 (0.140 / 80)	0.294 (0.113 / 100)
**Cystine**	4.1, 3.4, 3.2	Quartet, Quartet, Quartet	0.111 (0.000 / 20)	0.078 (0.043 / 40)	0.213 (0.177 / 20)	0.069 (0.026 / 40)	0.018 (0.000 / 10)

**N-Formyl-L-methionine**	8.1, 4.4, 2.5, 2.1, 2.0	Singlet, Quartet, Multiplet, Multiplet, Multiplet	0.320 (0.116 / 60)	0.045 (0.014 / 50)	0.213 (0.069 / 100)	0.108 (0.067 / 80)	0.048 (0.012 / 70)
**3-Hydroxymandelic acid**	7.3, 7.0, 6.9, 6.9	Triplet, Doublet, Triplet, Quartet	0.448 (0.015 / 40)	0.086 (0.033 / 80)	0.372 (0.077 / 90)	0.334 (0.058 / 80)	0.097 (0.047 / 70)
**Capric acid**	2.2, 1.5, 1.3, 0.9	Singlet, Singlet, Singlet, Multiplet	0.504 (0.079 / 40)	0.301 (0.113 / 60)	0.448 (0.149 / 40)	0.294 (0.000 / 20)	0.301 (0.124 / 20)
**Threonine**	4.2, 3.6, 1.3	Multiplet, Doublet, Doublet	0.227 (0.131 / 80)	0.041 (0.000 / 10)	0.133 (0.040 / 100)	0.317 (0.089 / 60)	0.048 (0.042 / 30)
**Beta-Alanine**	3.2, 2.6	Triplet, Triplet	0.094 (0.023 / 40)	0.036 (0.020 / 80)	0.070 (0.017 / 50)	0.133 (0.063 / 40)	0.024 (0.011 / 30)
**Alpha-D-Glucose**	5.2, 4.7, 3.9, 3.9, 3.8, 3.8, 3.7, 3.5, 3.5, 3.4, 3.2	Doublet, Doublet, Doublet, Doublet, Multiplet, Triplet, Multiplet, Quartet, Multiplet, Multiplet, Quartet	5.696 (3.028 / 80)	0.460 (0.288 / 50)	0.576 (0.638 / 90)	2.892 (1.096 / 80)	0.719 (0.405 / 70)
**Desaminotyrosine**	7.2, 6.8, 2.8, 2.4	Doublet, Doublet, Triplet, Quartet	0.014 (0.000 / 20)	0.004 (0.002 / 20)	0.019 (0.006 / 70)	0.011 (0.003 / 40)	0.004 (0.001 / 50)
**Xanthine**	7.9	Singlet	0.325 (0.184 / 60)	0.124 (0.040 / 100)	0.176 (0.126 / 100)	0.234 (0.153 / 100)	0.153 (0.065 / 90)
**Indoleacetic acid**	7.6, 7.5, 7.2, 7.2, 3.6	Doublet, Doublet, Multiplet, Triplet, Singlet	0.017 (0.018 / 80)	0.012 (0.010 / 80)	0.022 (0.024 / 60)	0.009 (0.000 / 20)	0.006 (0.001 / 30)
**Glycine**	3.5	Singlet	0.242 (0.096 / 60)	0.019 (0.008 / 90)	0.124 (0.056 / 90)	0.261 (0.181 / 80)	0.023 (0.013 / 90)
**3-Hydroxyisovaleric acid**	2.3, 1.3	Singlet, Singlet	0.072 (0.059 / 40)	0.074 (0.019 / 90)	0.156 (0.041 / 80)	0.157 (0.050 / 60)	0.038 (0.026 / 90)
**Putrescine**	3.0, 1.8	Multiplet, Multiplet	0.067 (0.000 / 40)	0.039 (0.000 / 10)	0.097 (0.000 / 10)	0.026 (0.001 / 40)	0.014 (0.007 / 60)
**Succinic acid**	2.4	Singlet	0.210 (0.103 / 100)	0.154 (0.076 / 100)	0.199 (0.083 / 100)	0.208 (0.105 / 100)	0.165 (0.072 / 90)
**Riboflavin**	7.4, 7.4, 4.5, 4.2, 4.0, 3.9, 3.8, 3.7, 2.3, 2.2	Singlet, Singlet, Singlet, Multiplet, Multiplet, Quartet, Quartet, Quartet, Singlet, Singlet	0.588 (0.304 / 100)	0.113 (0.058 / 100)	0.329 (0.132 / 100)	0.359 (0.270 / 100)	0.074 (0.030 / 90)
**N-Acetyl-L-alanine**	4.1, 2.0, 1.3	Multiplet, Singlet, Doublet	0.055 (0.000 / 20)	0.032 (0.015 / 40)	0.054 (0.013 / 20)	0.024 (0.000 / 20)	0.013 (0.021 / 70)
**N-Acetylserotonin**	9.9, 7.9, 7.4, 7.2, 7.1, 6.8, 3.5, 2.9, 1.9	Singlet, Singlet, Doublet, Doublet, Doublet, Quartet, Quartet, Triplet, Singlet	0.051 (0.005 / 80)	0.016 (0.011 / 60)	0.049 (0.009 / 80)	0.006 (0.000 / 20)	0.007 (0.006 / 80)
**Pantothenic acid**	4.0, 3.5, 3.5, 3.4, 3.4, 3.4, 2.4, 0.9, 0.9	Singlet, Singlet, Singlet, Quartet, Singlet, Singlet, Triplet, Singlet, Singlet	0.110 (0.062 / 100)	0.073 (0.039 / 100)	0.091 (0.053 / 100)	0.084 (0.037 / 100)	0.062 (0.019 / 100)
**m-Cresol**	7.2, 6.8, 6.8, 6.7, 2.3	Triplet, Doublet, Triplet, Quartet, Singlet	0.058 (0.008 / 40)	0.017 (0.012 / 40)	0.046 (0.008 / 50)	0.037 (0.010 / 60)	0.008 (0.002 / 70)
**Valeric acid**	2.2, 1.5, 1.3, 0.9	Triplet, Multiplet, Multiplet, Triplet	3.050 (0.000 / 20)	1.560 (0.206 / 40)	4.654 (2.348 / 40)	1.741 (0.000 / 20)	0.448 (0.438 / 20)
**Glyceraldehyde**	9.7, 3.7, 3.6	Singlet, Quartet, Multiplet	0.227 (0.000 / 20)	0.159 (0.032 / 90)	0.177 (0.026 / 60)	0.301 (0.075 / 40)	0.157 (0.044 / 80)
**Gamma-Butyrolactone**	4.3, 2.5, 2.2	Triplet, Multiplet, Multiplet	0.055 (0.000 / 20)	0.015 (0.002 / 30)	0.033 (0.000 / 10)	0.035 (0.011 / 60)	0.020 (0.009 / 20)
**Cyclic AMP**	8.2, 6.2, 4.5, 4.3	Doublet, Singlet, Multiplet, Multiplet	0.013 (0.000 / 20)	0.004 (0.001 / 60)	0.013 (0.003 / 50)	0.012 (0.004 / 60)	0.004 (0.000 / 10)
**N-Acetylmannosamine**	5.1, 5.0, 4.4, 4.3, 4.0, 3.9, 3.8, 3.6, 3.5, 3.5, 3.5, 3.4, 2.1, 2.0	Doublet, Doublet, Quartet, Quartet, Quartet, Doublet, Multiplet, Triplet, Singlet, Singlet, Singlet, Multiplet, Singlet, Singlet	1.981 (0.633 / 40)	0.142 (0.155 / 90)	0.427 (0.297 / 60)	0.952 (0.147 / 60)	0.075 (0.069 / 80)
**Argininosuccinic acid**	6.5, 4.2, 3.8, 3.3, 2.8, 2.8, 2.5, 2.5, 2.5, 1.9, 1.7	Singlet, Quartet, Triplet, Triplet, Doublet, Doublet, Singlet, Doublet, Singlet, Multiplet, Multiplet	1.402 (0.453 / 60)	0.343 (0.141 / 70)	0.979 (0.191 / 60)	0.346 (0.170 / 100)	0.236 (0.063 / 90)
**Citramalic acid**	2.8, 2.7, 2.5, 2.5, 2.2, 1.3	Singlet, Singlet, Singlet, Singlet, Singlet, Singlet	1.462 (0.000 / 20)	0.907 (0.318 / 70)	0.810 (0.321 / 50)	0.286 (0.221 / 100)	0.181 (0.069 / 90)

**Ureidosuccinic acid**	4.2, 2.7, 2.6, 2.5, 2.4	Triplet, Doublet, Doublet, Triplet, Singlet	1.646 (0.879 / 100)	0.183 (0.096 / 40)	2.014 (0.368 / 60)	0.609 (0.300 / 80)	0.142 (0.107 / 20)
**2-Furoylglycine**	7.7, 7.2, 6.6, 3.9	Doublet, Doublet, Quartet, Doublet	0.887 (0.572 / 60)	0.123 (0.040 / 60)	0.403 (0.241 / 40)	0.143 (0.048 / 80)	0.107 (0.028 / 50)
**Isovalerylglycine**	3.8, 2.2, 2.0, 0.9	Doublet, Doublet, Multiplet, Doublet	0.247 (0.103 / 60)	0.038 (0.010 / 60)	0.118 (0.041 / 80)	0.180 (0.111 / 80)	0.035 (0.009 / 60)
**Xanthosine**	7.9, 5.8, 4.4, 4.3, 3.9, 3.9, 3.8, 3.8	Singlet, Doublet, Quartet, Quartet, Doublet, Doublet, Doublet, Doublet	0.015 (0.004 / 40)	0.011 (0.005 / 50)	0.031 (0.017 / 70)	0.033 (0.018 / 60)	0.014 (0.011 / 60)
**Hydrocinnamic acid**	7.4, 7.3, 2.9, 2.5	Triplet, Multiplet, Triplet, Triplet	0.099 (0.022 / 100)	0.035 (0.011 / 90)	0.074 (0.013 / 90)	0.078 (0.016 / 100)	0.037 (0.010 / 90)
**Gulonic acid**	3.7, 3.7, 3.6, 3.6	Multiplicity, Multiplicity, Singlet, Multiplet	3.278 (1.476 / 40)	0.857 (0.232 / 60)	2.693 (0.426 / 40)	2.314 (0.392 / 60)	0.755 (0.118 / 90)
**Biotin**	4.3, 4.1, 3.1, 2.8, 2.6, 2.6, 2.2, 1.6, 1.5, 1.3	Quartet, Multiplet, Multiplet, Quartet, Singlet, Singlet, Triplet, Multiplet, Multiplet, Multiplet	1.810 (0.762 / 100)	0.594 (0.304 / 100)	0.927 (0.465 / 100)	0.151 (0.069 / 60)	0.129 (0.069 / 90)
**5-Methylcytidine**	7.7, 5.9, 4.3, 4.2, 4.1, 3.9, 3.9, 3.8, 2.0	Doublet, Doublet, Quartet, Triplet, Multiplet, Doublet, Doublet, Quartet, Doublet	0.046 (0.000 / 20)	0.051 (0.021 / 70)	0.095 (0.027 / 20)	0.030 (0.012 / 100)	0.033 (0.012 / 90)
**3-Hydroxyphenylacetic acid**	7.2, 6.9, 6.8, 3.5	Triplet, Doublet, Multiplet, Singlet	0.093 (0.030 / 100)	0.024 (0.010 / 70)	0.045 (0.021 / 90)	0.053 (0.001 / 40)	0.024 (0.007 / 80)
**L-3-Phenyllactic acid**	7.4, 7.3, 4.3, 3.1, 3.1, 2.9	Multiplet, Multiplet, Quartet, Doublet, Doublet, Quartet	0.057 (0.014 / 40)	0.011 (0.007 / 60)	0.040 (0.006 / 30)	0.037 (0.000 / 20)	0.018 (0.015 / 60)
**1-Methyladenosine**	8.4, 8.4, 8.2, 8.1, 6.1, 6.0, 4.4, 4.3, 3.9, 3.9	Singlet, Singlet, Singlet, Singlet, Doublet, Doublet, Multiplet, Multiplet, Multiplet, Multiplet	0.137 (0.076 / 80)	0.015 (0.005 / 70)	0.058 (0.037 / 80)	0.019 (0.003 / 80)	0.037 (0.022 / 80)
**4-Hydroxyproline**	4.3, 3.5, 3.4, 3.3, 2.4, 2.1	Quartet, Quartet, Doublet, Doublet, Multiplet, Multiplet	0.130 (0.070 / 80)	0.033 (0.016 / 90)	0.100 (0.031 / 100)	0.020 (0.000 / 20)	0.016 (0.005 / 80)
**Picolinic acid**	8.6, 8.0, 7.9, 7.5	Doublet, Multiplet, Multiplet, Multiplet	0.081 (0.042 / 60)	0.015 (0.004 / 40)	0.035 (0.025 / 40)	0.019 (0.000 / 20)	0.016 (0.005 / 20)
**Alanine**	3.8, 1.5	Quartet, Doublet	0.458 (0.246 / 80)	0.159 (0.054 / 100)	0.261 (0.088 / 100)	0.440 (0.233 / 100)	0.118 (0.033 / 90)
**Arabinose**	5.3, 5.2, 4.5, 4.1, 4.0, 4.0, 3.9, 3.8, 3.7, 3.5	Doublet, Quartet, Doublet, Multiplet, Multiplet, Multiplet, Multiplet, Multiplet, Multiplet, Quartet	0.278 (0.129 / 100)	0.050 (0.011 / 50)	0.164 (0.028 / 90)	0.662 (0.107 / 60)	0.147 (0.053 / 50)
**Formic acid**	8.4	Singlet	0.044 (0.022 / 60)	0.088 (0.027 / 80)	0.034 (0.017 / 100)	0.038 (0.022 / 80)	0.091 (0.026 / 70)
**Mevalonic acid**	4.5, 4.5, 2.7, 2.7, 2.6, 2.6, 2.0, 1.9, 1.4	Multiplet, Multiplet, Singlet, Singlet, Doublet, Doublet, Multiplet, Multiplet, Singlet	0.220 (0.058 / 40)	0.053 (0.018 / 20)	0.121 (0.021 / 30)	0.128 (0.039 / 80)	0.036 (0.017 / 90)
**Indolelactic acid**	7.7, 7.5, 7.2, 7.2, 4.3, 3.3, 3.2, 3.1	Doublet, Doublet, Multiplet, Multiplet, Quartet, Doublet, Doublet, Quartet	0.024 (0.014 / 80)	0.022 (0.014 / 70)	0.026 (0.022 / 90)	0.010 (0.000 / 20)	0.020 (0.008 / 40)
**Methylsuccinic acid**	2.6, 2.5, 2.1, 2.1, 2.1, 1.1	Multiplet, Quartet, Singlet, Doublet, Singlet, Doublet	0.085 (0.035 / 60)	0.007 (0.000 / 20)	0.043 (0.019 / 50)	0.040 (0.000 / 20)	0.009 (0.009 / 50)
**Glucose 6-phosphate**	5.2, 4.6, 4.0, 4.0, 3.9, 3.9, 3.7, 3.6, 3.5, 3.3	Doublet, Doublet, Multiplet, Quartet, Multiplet, Doublet, Triplet, Multiplet, Multiplet, Quartet	0.223 (0.160 / 80)	0.094 (0.030 / 80)	0.163 (0.128 / 100)	0.081 (0.016 / 60)	0.054 (0.008 / 50)
**4-Methylcatechol**	6.8, 6.8, 6.7, 2.2	Doublet, Doublet, Quartet, Singlet	0.131 (0.000 / 20)	0.012 (0.014 / 90)	0.047 (0.023 / 90)	0.034 (0.007 / 100)	0.009 (0.006 / 70)
**Dimethylsulfide**	2.1	Singlet	2.280 (0.586 / 80)	0.233 (0.050 / 30)	1.324 (0.426 / 100)	0.695 (0.223 / 100)	0.207 (0.020 / 60)
**Nicotinic acid**	8.9, 8.6, 8.3, 7.5	Doublet, Quartet, Quartet, Quartet	0.399 (0.157 / 80)	0.160 (0.027 / 80)	0.331 (0.090 / 100)	0.405 (0.137 / 100)	0.164 (0.026 / 70)

**Gluconic acid**	4.1, 4.0, 3.8, 3.8, 3.7	Doublet, Triplet, Multiplet, Multiplet, Multiplet	1.082 (0.316 / 80)	0.167 (0.070 / 100)	0.669 (0.312 / 100)	0.837 (0.051 / 40)	0.155 (0.059 / 90)
**N-Butyrylglycine**	3.9, 2.3, 1.6, 0.9	Doublet, Triplet, Multiplet, Triplet	0.243 (0.016 / 40)	0.040 (0.000 / 10)	0.257 (0.061 / 90)	0.067 (0.000 / 20)	0.048 (0.000 / 10)
**Theophylline**	8.0, 3.5, 3.3	Singlet, Singlet, Singlet	0.006 (0.000 / 20)	0.005 (0.003 / 30)	0.021 (0.009 / 40)	0.003 (0.001 / 100)	0.196 (0.432 / 60)
**Gabapentin**	3.0, 2.4, 1.5, 1.4	Singlet, Singlet, Multiplet, Multiplet	0.331 (0.228 / 100)	0.019 (0.000 / 10)	0.158 (0.071 / 70)	0.057 (0.046 / 100)	0.025 (0.007 / 50)
**Lactic acid**	4.1, 1.3	Quartet, Doublet	0.151 (0.052 / 40)	0.040 (0.019 / 30)	0.105 (0.027 / 40)	0.137 (0.000 / 20)	0.093 (0.031 / 20)
**Hydroxyacetone**	4.4, 2.1	Singlet, Singlet	0.160 (0.077 / 100)	0.034 (0.018 / 90)	0.094 (0.030 / 80)	0.173 (0.058 / 60)	0.060 (0.021 / 90)
**2-Isopropylmalic acid**	2.7, 2.7, 2.6, 2.5, 1.9, 0.9, 0.9	Singlet, Singlet, Singlet, Singlet, Triplet, Doublet, Doublet	5.971 (2.668 / 60)	1.785 (1.351 / 60)	4.081 (1.655 / 90)	0.426 (0.106 / 100)	0.357 (0.243 / 100)
**N-Phenylacetylphenylalanine**	7.7, 7.3, 7.3, 7.3, 7.3, 7.3, 7.3, 7.2, 7.2, 7.1, 7.1, 4.5, 3.6, 3.5, 3.2, 2.9	Doublet, Multiplet, Multiplet, Multiplet, Multiplet, Multiplet, Multiplet, Doublet, Doublet, Multiplet, Multiplet, Multiplet, Doublet, Doublet, Quartet, Quartet	0.031 (0.010 / 40)	0.026 (0.008 / 90)	0.052 (0.000 / 10)	0.016 (0.005 / 40)	0.016 (0.003 / 60)
**Hydroxyphenyllactic acid**	7.2, 6.8, 4.3, 3.0, 2.9	Doublet, Multiplet, Quartet, Quartet, Multiplet	0.329 (0.088 / 80)	0.079 (0.023 / 70)	0.182 (0.032 / 70)	0.172 (0.000 / 20)	0.072 (0.028 / 80)
**Cytidine monophosphate**	8.1, 6.1, 6.0, 4.3, 4.2, 4.0, 4.0	Doublet, Doublet, Doublet, Multiplet, Doublet, Multiplet, Multiplet	0.016 (0.003 / 60)	0.012 (0.002 / 50)	0.021 (0.004 / 80)	0.022 (0.008 / 40)	0.014 (0.004 / 60)
**N-Acetyl-L-tyrosine**	7.7, 7.2, 6.9, 4.4, 3.1, 2.8, 1.9	Doublet, Doublet, Doublet, Multiplet, Quartet, Quartet, Singlet	0.579 (0.158 / 60)	0.171 (0.055 / 70)	0.369 (0.089 / 60)	0.195 (0.090 / 40)	0.124 (0.042 / 80)
**Butanone**	2.6, 2.2, 1.0	Quartet, Singlet, Triplet	0.196 (0.028 / 60)	0.037 (0.020 / 60)	0.064 (0.044 / 70)	0.065 (0.000 / 20)	0.020 (0.005 / 40)
**Glucose**	5.2, 4.6, 3.9, 3.9, 3.8, 3.8, 3.7, 3.5, 3.5, 3.4, 3.2	Doublet, Doublet, Doublet, Doublet, Multiplet, Triplet, Multiplet, Quartet, Multiplet, Multiplet, Quartet	0.328 (0.306 / 60)	0.057 (0.030 / 90)	0.197 (0.155 / 100)	0.366 (0.098 / 100)	0.065 (0.014 / 60)
**Isoferulic acid**	7.3, 7.3, 7.1, 7.0, 6.4, 6.3, 3.9	Singlet, Singlet, Triplet, Doublet, Singlet, Singlet, Singlet	0.006 (0.000 / 20)	0.013 (0.005 / 40)	0.022 (0.010 / 70)	0.005 (0.002 / 60)	0.035 (0.040 / 30)
**Cytidine**	7.8, 6.0, 5.9, 4.3, 4.2, 4.1, 3.9, 3.9, 3.8	Doublet, Doublet, Doublet, Quartet, Triplet, Multiplet, Doublet, Doublet, Quartet	- (- / 0)	0.013 (0.002 / 50)	0.019 (0.000 / 10)	0.046 (0.000 / 20)	0.019 (0.008 / 60)
**Ortho-Hydroxyphenylacetic acid**	7.2, 6.9, 3.6	Multiplet, Multiplet, Singlet	- (- / 0)	0.078 (0.022 / 40)	0.214 (0.035 / 30)	0.111 (0.043 / 60)	0.048 (0.012 / 70)
**Rhamnose**	5.1, 3.9, 3.9, 3.8, 3.6, 3.4, 3.4, 3.4, 1.3	Doublet, Multiplet, Quartet, Quartet, Quartet, Singlet, Singlet, Multiplet, Quartet	- (- / 0)	0.018 (0.019 / 80)	0.016 (0.000 / 10)	0.129 (0.055 / 60)	0.056 (0.016 / 50)
**Alpha-Hydroxyisobutyric acid**	1.3	Singlet	- (- / 0)	0.011 (0.000 / 10)	0.015 (0.005 / 30)	0.050 (0.014 / 60)	0.014 (0.006 / 40)
**p-Aminobenzoic acid**	7.7, 6.8	Doublet, Doublet	- (- / 0)	0.007 (0.002 / 30)	0.012 (0.000 / 10)	0.017 (0.000 / 20)	0.007 (0.001 / 20)
**2-Hydroxybutyric acid**	4.0, 0.9	Quartet, Triplet	- (- / 0)	0.105 (0.000 / 10)	0.575 (0.477 / 40)	0.353 (0.000 / 20)	0.163 (0.020 / 20)
**Beta-N-Acetylglucosamine**	5.2, 3.9, 3.9, 3.8, 3.7, 3.5, 3.5, 3.5, 2.0	Doublet, Doublet, Multiplet, Multiplet, Quartet, Triplet, Singlet, Multiplet, Singlet	- (- / 0)	0.170 (0.072 / 80)	0.475 (0.267 / 80)	0.451 (0.266 / 100)	0.158 (0.099 / 90)
**Tartaric acid**	4.3	Singlet	- (- / 0)	0.075 (0.026 / 60)	0.238 (0.000 / 10)	0.152 (0.000 / 20)	0.092 (0.007 / 20)
**Hydroxyoctanoic acid**	4.0, 1.7, 1.6, 1.3, 0.9	Quartet, Multiplet, Multiplet, Multiplet, Multiplet	- (- / 0)	0.081 (0.020 / 70)	0.194 (0.074 / 30)	0.186 (0.000 / 20)	0.078 (0.017 / 60)

**Methionine**	3.9, 2.6, 2.2, 2.1, 2.1	Quartet, Triplet, Multiplet, Singlet, Multiplet	- (- / 0)	0.009 (0.006 / 20)	0.040 (0.006 / 40)	0.029 (0.007 / 80)	0.002 (0.000 / 10)
**S-Adenosylhomocysteine**	8.4, 8.3, 6.1, 4.9, 4.4, 4.3, 3.8, 3.1, 3.0, 2.7, 2.1, 2.1	Singlet, Singlet, Doublet, Triplet, Triplet, Multiplet, Quartet, Quartet, Quartet, Triplet, Multiplet, Multiplet	- (- / 0)	0.016 (0.002 / 60)	0.062 (0.023 / 90)	0.047 (0.000 / 20)	0.029 (0.000 / 10)
**Acetylcarnitine**	5.6, 3.8, 3.6, 3.6, 3.2, 2.6, 2.6, 2.5, 2.1	Quartet, Quartet, Singlet, Singlet, Singlet, Doublet, Doublet, Quartet, Singlet	0.178 (0.000 / 20)	0.049 (0.014 / 70)	0.114 (0.019 / 20)	0.039 (0.012 / 40)	0.023 (0.022 / 80)
**N-Acetylgalactosamine**	5.2, 4.1, 4.0, 3.9, 3.7, 2.0	Doublet, Multiplet, Multiplet, Multiplet, Multiplet, Quartet	- (- / 0)	0.095 (0.000 / 10)	0.582 (0.144 / 40)	0.095 (0.000 / 20)	0.159 (0.058 / 50)
**Methylcysteine**	3.9, 3.1, 3.1, 3.0, 2.2	Quartet, Doublet, Doublet, Quartet, Singlet	- (- / 0)	0.183 (0.208 / 100)	0.766 (0.716 / 60)	0.546 (0.164 / 80)	0.066 (0.053 / 90)
**1,3,7-Trimethyluric acid**	3.4, 3.3, 3.2	Singlet, Singlet, Singlet	0.390 (0.015 / 40)	- (- / 0)	0.049 (0.023 / 50)	0.044 (0.030 / 100)	0.037 (0.041 / 50)
**Homogentisic acid**	6.8, 6.7, 3.5	Triplet, Multiplet, Singlet	0.393 (0.002 / 40)	- (- / 0)	0.183 (0.032 / 50)	0.082 (0.000 / 20)	0.073 (0.018 / 70)
**Threitol**	3.7, 3.7, 3.6	Multiplet, Doublet, Quartet	0.714 (0.264 / 40)	- (- / 0)	0.306 (0.000 / 10)	0.397 (0.196 / 60)	0.061 (0.044 / 20)
**Glycyl-glycine**	3.8, 3.8	Singlet, Singlet	0.154 (0.039 / 40)	- (- / 0)	0.080 (0.016 / 30)	0.102 (0.050 / 100)	0.012 (0.002 / 30)
**2-Aminoisobutyric acid**	1.5	Singlet	0.059 (0.000 / 20)	- (- / 0)	0.016 (0.006 / 30)	0.011 (0.006 / 100)	0.029 (0.063 / 80)
**Methylamine**	2.6	Singlet	0.160 (0.001 / 40)	- (- / 0)	0.114 (0.065 / 80)	0.162 (0.043 / 100)	0.011 (0.008 / 70)
**Adenosine monophosphate**	8.6, 8.2, 6.1, 4.5, 4.4, 4.0	Singlet, Doublet, Quartet, Doublet, Quartet	0.023 (0.013 / 60)	- (- / 0)	0.010 (0.000 / 10)	0.006 (0.000 / 20)	0.007 (0.001 / 30)
**Pyruvic acid**	2.4	Singlet	0.046 (0.000 / 20)	- (- / 0)	0.049 (0.005 / 20)	0.018 (0.014 / 100)	0.011 (0.004 / 40)
**Guanidinosuccinic acid**	4.2, 2.8, 2.8, 2.5, 2.5	Quartet, Doublet, Doublet, Singlet, Triplet	0.030 (0.000 / 20)	- (- / 0)	0.010 (0.000 / 10)	0.033 (0.014 / 40)	0.024 (0.010 / 30)
**Cysteine-S-sulfate**	4.2, 3.7, 3.7, 3.5	Quartet, Doublet, Doublet, Quartet	0.587 (0.335 / 40)	- (- / 0)	0.715 (0.000 / 10)	0.716 (0.000 / 20)	0.038 (0.030 / 20)
**dCMP**	8.0, 6.3, 6.1, 4.6, 4.2, 4.0, 2.4, 2.3	Doublet, Triplet, Doublet, Multiplet, Multiplet, Multiplet, Multiplet, Multiplet	0.166 (0.000 / 20)	- (- / 0)	0.129 (0.000 / 10)	0.169 (0.000 / 20)	0.020 (0.000 / 10)
**2-Hydroxy-3-methylbutyric acid**	3.9, 2.0, 1.0, 0.8	Doublet, Multiplet, Doublet, Doublet	3.252 (0.000 / 20)	- (- / 0)	1.575 (0.000 / 10)	2.796 (1.273 / 40)	0.935 (0.215 / 20)
**3-Methyl-2-oxovaleric acid**	2.9, 1.7, 1.5, 1.1, 0.9	Multiplet, Multiplet, Multiplet, Doublet, Triplet	1.120 (0.362 / 100)	- (- / 0)	0.500 (0.319 / 70)	1.417 (0.677 / 60)	0.004 (0.000 / 10)
**Methanol**	3.3	Singlet	0.263 (0.120 / 100)	- (- / 0)	0.665 (0.143 / 100)	0.056 (0.103 / 100)	0.001 (0.000 / 30)
**Trimethylamine N-oxide**	3.2	Singlet	0.006 (0.000 / 20)	- (- / 0)	0.011 (0.010 / 20)	0.003 (0.001 / 100)	0.001 (0.001 / 40)
**Methylguanidine**	3.4, 2.8	Singlet, Singlet	0.193 (0.163 / 40)	- (- / 0)	0.134 (0.075 / 50)	0.032 (0.020 / 80)	0.019 (0.009 / 40)
**Citric acid**	2.7, 2.6, 2.5, 2.5	Singlet, Singlet, Singlet, Singlet	0.043 (0.000 / 20)	- (- / 0)	0.057 (0.012 / 80)	0.007 (0.004 / 80)	0.007 (0.003 / 70)
**Ketoisovaleric acid**	3.0, 1.1	Multiplet, Doublet	0.044 (0.026 / 80)	- (- / 0)	0.039 (0.017 / 80)	0.052 (0.029 / 40)	0.098 (0.000 / 10)
**Trehalose**	5.2, 3.9, 3.8, 3.6, 3.4	Doublet, Doublet, Multiplet, Quartet, Triplet	0.093 (0.035 / 80)	- (- / 0)	0.093 (0.059 / 50)	0.177 (0.018 / 40)	- (- / 0)
**Epicatechin**	7.0, 6.9, 6.1, 6.1, 4.3, 2.9, 2.8, 2.7	Doublet, Multiplet, Doublet, Doublet, Multiplet, Multiplet, Doublet, Doublet	0.120 (0.040 / 40)	0.060 (0.032 / 50)	- (- / 0)	0.104 (0.057 / 40)	0.111 (0.062 / 80)
**Ribothymidine**	7.7, 5.9, 4.3, 4.2, 4.1, 3.9,	Doublet, Doublet, Triplet, Triplet, Multiplet, Doublet,	0.048 (0.000 / 20)	0.009 (0.003 / 50)	- (- / 0)	0.012 (0.007 / 80)	0.117 (0.250 / 70)

	3.9, 3.8, 1.9, 1.9	Doublet, Quartet, Doublet, Doublet					
**Uridine 5'-monophosphate**	8.1, 6.0, 4.4, 4.3, 4.0, 4.0	Doublet, Quartet, Triplet, Triplet, Multiplet, Multiplet	0.030 (0.000 / 20)	0.021 (0.000 / 10)	- (- / 0)	0.032 (0.000 / 20)	0.020 (0.000 / 10)
**Phosphorylcholine**	4.2, 3.6, 3.2	Multiplet, Multiplet, Singlet	0.343 (0.000 / 20)	0.028 (0.000 / 10)	- (- / 0)	0.028 (0.000 / 20)	0.006 (0.000 / 10)
**Sarcosine**	3.6, 2.7	Singlet, Singlet	0.023 (0.000 / 20)	0.011 (0.000 / 10)	- (- / 0)	0.010 (0.005 / 100)	0.007 (0.003 / 90)
**Hypoxanthine**	8.2, 8.2	Singlet, Singlet	0.040 (0.000 / 20)	0.048 (0.022 / 20)	- (- / 0)	0.007 (0.000 / 20)	0.033 (0.024 / 30)
**1-Methylguanosine**	8.0, 5.9, 4.4, 4.2, 3.9, 3.8, 3.5	Singlet, Doublet, Quartet, Triplet, Quartet, Quartet, Singlet	0.029 (0.002 / 40)	0.037 (0.000 / 10)	- (- / 0)	0.014 (0.006 / 60)	0.021 (0.011 / 40)
**Indole**	7.7, 7.5, 7.2, 7.1, 6.6	Doublet, Doublet, Multiplet, Multiplet, Triplet	0.128 (0.000 / 20)	0.025 (0.005 / 70)	0.050 (0.000 / 10)	0.023 (0.000 / 20)	0.029 (0.013 / 60)
**Pyrimidine**	9.1, 8.8, 7.6	Singlet, Doublet, Multiplet	0.048 (0.000 / 20)	0.042 (0.000 / 10)	0.042 (0.006 / 30)	- (- / 0)	0.040 (0.000 / 10)
**Ureidopropionic acid**	3.3, 2.4	Quartet, Triplet	0.170 (0.000 / 20)	0.060 (0.000 / 10)	0.147 (0.000 / 10)	- (- / 0)	0.029 (0.009 / 60)
**N-Acetylglutamine**	8.0, 706, 6.8, 4.2, 2.3, 2.3, 2.1, 2.0, 1.9	Doublet, Singlet, Singlet, Multiplet, Multiplet, Multiplet, Multiplet, Singlet, Multiplet	0.078 (0.038 / 80)	0.032 (0.013 / 30)	0.076 (0.043 / 20)	- (- / 0)	0.005 (0.000 / 10)
**Sedoheptulose**	4.0, 3.9, 3.8, 3.8, 3.6	Quartet, Multiplet, Singlet, Quartet, Doublet	0.327 (0.009 / 60)	0.066 (0.017 / 50)	0.232 (0.040 / 20)	0.367 (0.000 / 20)	0.052 (0.009 / 50)
**Tryptamine**	7.7, 7.5, 7.3, 7.3, 7.2, 3.3, 3.2	Doublet, Doublet, Singlet, Multiplet, Multiplet, Triplet, Triplet	0.080 (0.039 / 80)	0.031 (0.009 / 80)	0.049 (0.027 / 90)	0.033 (0.000 / 20)	0.016 (0.008 / 90)
**Kynurenine**	7.8, 7.4, 6.9, 6.8, 4.1, 3.7	Quartet, Multiplet, Quartet, Multiplet, Quartet, Multiplet	1.569 (0.641 / 80)	0.372 (0.215 / 90)	0.970 (0.299 / 100)	- (- / 0)	0.321 (0.116 / 60)
**Hexadecanedioic acid**	2.2, 1.9, 1.9, 1.7, 1.6, 1.4, 1.3, 1.1	Multiplet, Multiplet, Multiplet, Multiplet, Multiplet, Multiplet, Multiplet, Multiplet	0.435 (0.170 / 60)	0.095 (0.034 / 100)	0.855 (0.359 / 80)	- (- / 0)	0.026 (0.000 / 10)
**3-Methylindole**	7.7, 7.5, 7.2, 7.2, 2.3	Doublet, Doublet, Multiplet, Multiplet, Doublet	0.020 (0.014 / 100)	0.010 (0.004 / 100)	0.029 (0.008 / 100)	- (- / 0)	0.012 (0.005 / 50)
**L-Dopa**	6.9, 6.8, 6.7, 3.9, 3.2, 3.1, 3.0	Doublet, Doublet, Quartet, Quartet, Doublet, Doublet, Quartet	0.033 (0.000 / 20)	0.024 (0.008 / 60)	0.014 (0.000 / 10)	- (- / 0)	0.025 (0.010 / 50)
**o-Cresol**	7.2, 7.1, 6.9, 2.2	Doublet, Multiplet, Multiplet, Singlet	0.149 (0.124 / 60)	0.127 (0.032 / 50)	0.143 (0.130 / 70)	- (- / 0)	0.063 (0.037 / 100)
**5'-Methylthioadenosine**	8.5, 8.4, 6.1, 4.4, 4.3, 2.9, 2.1	Singlet, Singlet, Doublet, Triplet, Multiplet, Multiplet, Singlet	0.003 (0.000 / 20)	0.002 (0.001 / 40)	0.002 (0.000 / 10)	- (- / 0)	0.053 (0.086 / 40)
**N6-Acetyl-L-lysine**	3.7, 3.2, 2.0, 1.9, 1.6, 1.4	Quartet, Quartet, Singlet, Multiplet, Multiplet, Multiplet	2.009 (0.000 / 20)	0.484 (0.293 / 50)	1.148 (0.160 / 40)	- (- / 0)	0.089 (0.024 / 40)
**Deoxyuridine**	7.8, 6.3, 5.9, 4.5, 4.1, 3.8, 3.8, 2.4	Doublet, Triplet, Doublet, Multiplet, Multiplet, Quartet, Quartet, Multiplet	0.050 (0.027 / 80)	0.016 (0.004 / 50)	0.041 (0.016 / 80)	- (- / 0)	0.019 (0.006 / 50)
**Mannose**	5.2, 3.9, 3.9, 3.8, 3.6, 3.6, 3.5, 3.4	Doublet, Multiplet, Quartet, Multiplet, Multiplet, Doublet, Singlet, Multiplet	0.802 (0.304 / 100)	0.126 (0.067 / 100)	0.387 (0.298 / 80)	- (- / 0)	0.124 (0.014 / 80)
**Mannose 6-phosphate**	5.2, 4.0, 3.9, 3.8, 3.7, 3.4	Singlet, Multiplet, Quartet, Triplet, Multiplet, Multiplet	0.076 (0.035 / 40)	0.066 (0.025 / 70)	0.171 (0.068 / 80)	- (- / 0)	0.424 (0.399 / 20)
**Cinnamic acid**	7.6, 7.4, 7.4, 6.5, 6.5	Multiplet, Multiplet, Singlet, Singlet, Singlet	0.257 (0.101 / 100)	0.042 (0.015 / 50)	0.123 (0.056 / 100)	- (- / 0)	0.026 (0.009 / 50)
**Taurodeoxycholic acid**	7.9, 4.0, 3.6, 3.6, 3.1, 2.3,	Triplet, Doublet, Multiplet, Quartet, Triplet, Multiplet,	0.048 (0.014 / 40)	0.018 (0.009 / 90)	0.030 (0.025 / 100)	- (- / 0)	0.019 (0.002 / 30)

	2.1, 1.9, 1.8, 1.7, 1.6, 1.6, 1.5, 1.4, 1.2, 1.0, 0.9, 0.7	Multiplet, Singlet, Singlet, Multiplet, Singlet, Doublet, Multiplet, Singlet, Multiplet, Doublet, Singlet, Singlet					
**Kynurenic acid**	7.9, 7.6, 7.5, 7.3, 6.7	Doublet, Multiplet, Doublet, Quartet, Singlet	0.039 (0.016 / 60)	0.013 (0.003 / 40)	0.031 (0.004 / 50)	- (- / 0)	0.014 (0.004 / 60)
**2-Oxohexane**	2.5, 2.2, 1.5, 1.3, 0.9	Triplet, Singlet, Multiplet, Multiplet, Triplet	0.584 (0.076 / 60)	0.170 (0.130 / 50)	0.472 (0.259 / 80)	- (- / 0)	0.095 (0.035 / 50)
**Caffeic acid**	7.3, 7.3, 7.1, 7.1, 6.9, 6.3, 6.3	Singlet, Singlet, Doublet, Quartet, Doublet, Singlet, Singlet	0.088 (0.020 / 60)	0.021 (0.002 / 30)	0.075 (0.026 / 40)	- (- / 0)	0.016 (0.001 / 40)
**4-Hydroxybenzoic acid**	7.8, 6.9	Doublet, Doublet	0.005 (0.000 / 20)	0.008 (0.005 / 20)	0.021 (0.004 / 40)	- (- / 0)	0.014 (0.000 / 10)
**Hydrochlorothiazide**	8.2, 7.1	Singlet, Singlet	0.663 (0.179 / 40)	0.169 (0.062 / 70)	0.230 (0.160 / 20)	- (- / 0)	0.113 (0.029 / 40)
**Tryptophan**	7.7, 7.5, 7.3, 7.3, 7.2, 4.0, 3.5, 3.5, 3.3	Doublet, Doublet, Singlet, Multiplet, Multiplet, Quartet, Doublet, Doublet, Quartet	0.053 (0.026 / 100)	0.024 (0.012 / 100)	0.044 (0.016 / 100)	- (- / 0)	0.016 (0.007 / 80)
**Glucuronic acid**	5.3, 4.6, 4.1, 4.1, 3.7, 3.6, 3.5, 3.3	Triplet, Singlet, Singlet, Singlet, Multiplet, Quartet, Multiplet, Triplet	0.172 (0.108 / 60)	0.019 (0.002 / 50)	0.075 (0.045 / 100)	- (- / 0)	0.045 (0.021 / 20)
**Deoxyinosine**	8.3, 8.2, 6.5, 4.2, 3.8, 3.8, 2.8, 2.6	Singlet, Singlet, Triplet, Multiplet, Quartet, Quartet, Multiplet, Multiplet	0.628 (0.000 / 20)	0.232 (0.038 / 30)	0.312 (0.000 / 10)	0.169 (0.000 / 20)	0.145 (0.077 / 50)
**2-Aminobenzoic acid**	7.7, 7.3, 6.9, 3.8	Quartet, Multiplet, Quartet, Multiplet	0.210 (0.000 / 20)	0.183 (0.041 / 70)	0.408 (0.102 / 50)	- (- / 0)	0.145 (0.030 / 50)
**Fumaric acid**	6.5	Singlet	0.017 (0.011 / 100)	0.002 (0.000 / 20)	0.014 (0.009 / 90)	0.036 (0.016 / 100)	- (- / 0)
**Uridine**	7.9, 5.9, 4.3, 4.2, 4.1, 3.9, 3.9, 3.8, 3.4	Doublet, Triplet, Triplet, Triplet, Multiplet, Doublet, Doublet, Quartet, Singlet	0.017 (0.000 / 20)	0.004 (0.000 / 10)	0.032 (0.000 / 20)	0.028 (0.000 / 20)	- (- / 0)
**Ribose 5-phosphate**	5.4, 5.2, 4.3, 4.2, 4.1, 3.9	Multiplet, Singlet, Multiplet, Multiplet, Multiplet, Multiplet	2.251 (0.449 / 40)	0.956 (0.000 / 10)	1.871 (0.712 / 50)	2.249 (0.646 / 60)	- (- / 0)
**Gallic acid**	7	Singlet	- (- / 0)	0.015 (0.008 / 50)	- (- / 0)	0.015 (0.001 / 40)	0.021 (0.009 / 80)
**Folic acid**	8.5, 8.0, 7.5, 6.4, 4.3, 4.2, 2.3, 2.2, 2.0	Singlet, Doublet, Doublet, Doublet, Multiplet, Singlet, Multiplet, Multiplet, Multiplet	0.015 (0.004 / 60)	- (- / 0)	0.016 (0.005 / 70)	- (- / 0)	0.015 (0.000 / 10)
**Suberic acid**	2.2, 1.5, 1.3	Triplet, Triplet, Multiplet	- (- / 0)	0.087 (0.011 / 30)	0.266 (0.014 / 20)	- (- / 0)	0.116 (0.000 / 10)
**Guanidoacetic acid**	3.8	Singlet	- (- / 0)	0.199 (0.027 / 20)	- (- / 0)	0.824 (0.089 / 40)	0.165 (0.034 / 70)
**Thymidine**	7.6, 6.3, 4.5, 4.0, 3.8, 3.8, 2.4, 2.4, 1.9	Doublet, Triplet, Quartet, Multiplet, Quartet, Quartet, Quartet, Quartet, Doublet	0.056 (0.000 / 20)	0.012 (0.003 / 20)	- (- / 0)	- (- / 0)	0.006 (0.003 / 70)
**Decanoylcarnitine**	5.6, 3.8, 3.6, 3.6, 3.2, 3.2, 2.6, 2.5, 2.4, 1.6, 1.3, 0.8	Multiplet, Quartet, Singlet, Singlet, Singlet, Doublet, Quartet, Quartet, Triplet, Multiplet, Multiplet, Triplet	- (- / 0)	- (- / 0)	0.063 (0.002 / 20)	0.028 (0.012 / 80)	0.021 (0.005 / 70)
**Aminocaproic acid**	3.0, 2.2, 1.7, 1.6, 1.4	Triplet, Triplet, Multiplet, Multiplet, Multiplet	- (- / 0)	0.227 (0.051 / 50)	0.696 (0.200 / 20)	- (- / 0)	0.126 (0.044 / 40)
**Dihydroorotic acid**	4.1, 3.0, 3.0, 2.8, 2.8	Triplet, Doublet, Doublet, Doublet, Doublet	1.380 (0.000 / 20)	0.225 (0.082 / 20)	0.909 (0.808 / 20)	- (- / 0)	- (- / 0)

**Biocytin**	4.6, 4.4, 3.7, 3.3, 3.2, 3.0, 2.8, 2.2, 1.9, 1.6, 1.4	Quartet, Multiplet, Triplet, Multiplet, Multiplet, Quartet, Doublet, Triplet, Multiplet, Multiplet, Multiplet	- (- / 0)	0.200 (0.077 / 30)	0.749 (0.000 / 10)	- (- / 0)	0.066 (0.028 / 50)
**Propyl alcohol**	3.6, 1.5, 0.9	Triplet, Multiplet, Triplet	- (- / 0)	0.115 (0.000 / 10)	0.646 (0.312 / 20)	- (- / 0)	0.165 (0.014 / 50)
**Salicyluric acid**	7.8, 7.5, 7.0, 4.0	Quartet, Multiplet, Quartet, Doublet	- (- / 0)	0.030 (0.000 / 10)	0.026 (0.008 / 20)	- (- / 0)	0.015 (0.007 / 30)
**3,7-Dimethyluric acid**	3.4, 3.4	Singlet, Singlet	0.252 (0.092 / 60)	- (- / 0)	- (- / 0)	0.635 (0.222 / 100)	0.048 (0.035 / 20)
**Deoxycholic acid glycine conjugate**	7.8, 4.0, 3.7, 3.6, 2.3, 2.2, 1.8, 1.5, 1.4, 1.3, 1.2, 1.1, 1.0, 0.9, 0.7	Trilpet, Singlet, Multiplet, Multiplet, Multiplet, Multiplet, Multiplet, Multiplet, Multiplet, Triplet, Doublet, Multiplet, Doublet, Singlet, Singlet	- (- / 0)	0.006 (0.003 / 50)	0.016 (0.009 / 70)	- (- / 0)	0.010 (0.005 / 20)
**Pelargonic acid**	2.2, 1.5, 1.3, 0.9	Triplet, Triplet, Multiplet, Multiplet	- (- / 0)	0.055 (0.011 / 20)	0.091 (0.006 / 20)	- (- / 0)	0.057 (0.004 / 20)
**Glyoxylic acid**	9.4, 5.1	Singlet, Singlet	- (- / 0)	0.002 (0.000 / 10)	- (- / 0)	0.049 (0.006 / 60)	0.072 (0.012 / 90)
**3-Methyladipic acid**	2.4, 2.2, 1.9, 1.6, 1.5, 0.9	Multiplet, Quartet, Multiplet, Multiplet, Multiplet, Doublet	0.534 (0.060 / 60)	- (- / 0)	0.692 (0.178 / 50)	0.305 (0.000 / 20)	- (- / 0)
**Geraniol**	5.4, 4.1, 2.1, 1.7, 1.7, 1.6	Multiplet, Triplet, Multiplet, Doublet, Singlet, Singlet	- (- / 0)	0.190 (0.012 / 20)	- (- / 0)	0.116 (0.037 / 80)	0.032 (0.010 / 90)
**Alpha-Lactose**	5.2, 4.4, 3.9, 3.9, 3.8, 3.6, 3.3	Doublet, Doublet, Multiplet, Doublet, Multiplet, Multiplet, Multiplet	- (- / 0)	0.038 (0.019 / 30)	0.212 (0.008 / 20)	0.199 (0.000 / 20)	- (- / 0)
**Propanal**	9.7, 2.6, 1.0	Triplet, Multiplet, Multiplet	- (- / 0)	0.007 (0.002 / 60)	0.009 (0.000 / 10)	- (- / 0)	0.092 (0.000 / 10)
**1-Butanol**	3.6, 1.5, 1.3, 0.9	Triplet, Multiplet, Multiplet, Triplet	- (- / 0)	0.017 (0.000 / 10)	0.064 (0.006 / 20)	- (- / 0)	0.025 (0.007 / 60)
**Chenodeoxycholic acid**	3.9, 3.5, 2.2, 2.1, 2.0, 1.9, 1.7, 1.5, 1.4, 1.3, 1.1, 1.0, 1.0, 1.0, 0.9, 0.7	Doublet, Multiplet, Multiplet, Multiplet, Multiplet, Multiplet, Multiplet, Multiplet, Multiplet, Multiplet, Multiplet, Doublet, Doublet, Doublet, Triplet, Singlet	- (- / 0)	0.097 (0.049 / 90)	0.201 (0.167 / 90)	- (- / 0)	0.085 (0.026 / 80)
**1-Methyluric acid**	3.3	Singlet	0.457 (0.000 / 20)	- (- / 0)	- (- / 0)	0.213 (0.128 / 60)	0.383 (0.000 / 10)
**N-Methylhydantoin**	4.1, 2.9	Singlet, Singlet	- (- / 0)	0.061 (0.000 / 10)	- (- / 0)	0.048 (0.014 / 80)	0.044 (0.021 / 80)
**Xanthurenic acid**	7.4, 7.2, 7.0, 6.7	Quartet, Triplet, Quartet, Singlet	- (- / 0)	0.009 (0.003 / 60)	0.016 (0.008 / 20)	- (- / 0)	0.010 (0.002 / 80)
**Maltotriose**	5.4, 5.2, 3.9, 3.9, 3.8, 3.7, 3.7, 3.6, 3.6, 3.6, 3.4, 3.4, 3.3	Triplet, Doublet, Multiplet, Quartet, Multiplet, Multiplet, Singlet, Multiplet, Singlet, Singlet, Singlet, Doublet, Quartet	- (- / 0)	0.076 (0.015 / 20)	0.193 (0.028 / 50)	- (- / 0)	0.307 (0.092 / 80)
**3-Aminoisobutanoic acid**	3.1, 3.0, 2.9, 2.6, 1.8, 1.2	Quartet, Quartet, Triplet, Multiplet, Singlet, Doublet	- (- / 0)	0.596 (0.000 / 10)	- (- / 0)	0.330 (0.089 / 60)	0.303 (0.000 / 10)
**Traumatic acid**	6.7, 5.8, 5.8, 2.2, 1.5, 1.4, 1.3	Multiplet, Triplet, Triplet, Multiplet, Triplet, Triplet, Doublet	0.027 (0.010 / 80)	0.007 (0.000 / 10)	0.021 (0.006 / 50)	- (- / 0)	- (- / 0)
**Carnosine**	8.1, 7.1, 4.5, 3.2, 3.2, 3.0, 2.7	Singlet, Singlet, Quartet, Multiplet, Doublet, Quartet, Multiplet	- (- / 0)	0.016 (0.000 / 10)	- (- / 0)	0.014 (0.004 / 60)	0.024 (0.013 / 20)
**Caproic acid**	2.2, 1.5, 1.3, 0.9	Triplet, Multiplet, Multiplet, Triplet	0.517 (0.000 / 20)	0.165 (0.048 / 20)	0.275 (0.114 / 30)	- (- / 0)	0.104 (0.015 / 20)

**Anserine**	8.3, 7.1, 4.5, 3.8, 3.2, 3.0, 2.7	Doublet, Doublet, Quartet, Singlet, Multiplet, Quartet, Multiplet	- (- / 0)	0.028 (0.000 / 10)	- (- / 0)	0.070 (0.041 / 100)	0.069 (0.018 / 30)
**Niacinamide**	8.9, 8.7, 8.2, 8.2, 7.6, 7.4	Doublet, Quartet, Multiplet, Multiplet, Multiplet, Multiplet	0.027 (0.025 / 100)	- (- / 0)	0.020 (0.015 / 30)	- (- / 0)	0.147 (0.138 / 20)
**Glycerol**	3.8, 3.6, 3.6	Multiplet, Quartet, Quartet, Quartet	0.639 (0.000 / 20)	0.280 (0.000 / 10)	1.744 (0.344 / 30)	- (- / 0)	- (- / 0)
**Norleucine**	3.7, 1.9, 1.4, 0.9	Quartet, Multiplet, Multiplet, Triplet	- (- / 0)	2.509 (0.361 / 20)	5.294 (0.014 / 20)	- (- / 0)	1.647 (0.444 / 30)
**Ethylmethylacetic acid**	2.3, 1.5, 1.1, 0.9	Multiplet, Multiplet, Doublet, Triplet	- (- / 0)	- (- / 0)	0.279 (0.118 / 50)	0.116 (0.015 / 40)	0.019 (0.000 / 10)
**Quinolinic acid**	8.4, 8.0, 7.4	Quartet, Quartet, Quartet	- (- / 0)	0.012 (0.002 / 40)	0.014 (0.003 / 30)	- (- / 0)	0.015 (0.003 / 50)
**Quinic acid**	4.1, 4.0, 3.5, 2.0, 2.0, 1.9, 1.9, 1.8	Quartet, Multiplet, Quartet, Multiplet, Multiplet, Singlet, Doublet, Singlet	1.043 (0.000 / 20)	- (- / 0)	- (- / 0)	0.259 (0.208 / 80)	0.095 (0.009 / 20)
**Isoleucine**	3.7, 2.0, 1.5, 1.2, 1.0, 0.9	Doublet, Multiplet, Multiplet, Multiplet, Doublet, Triplet	1.015 (0.000 / 20)	0.061 (0.014 / 70)	0.156 (0.053 / 90)	0.974 (0.000 / 20)	0.054 (0.014 / 80)
**4-Heptanone**	2.5, 1.5, 0.9	Triplet, Doublet, Triplet	0.832 (0.000 / 20)	0.249 (0.058 / 50)	0.493 (0.039 / 30)	- (- / 0)	0.005 (0.000 / 10)
**Glycylproline**	4.3, 4.3, 3.9, 3.9, 3.6, 3.6, 3.6, 3.5, 3.5, 2.3, 2.2, 2.1, 2.0, 2.0, 1.9, 1.9, 1.8	Quartet, Quartet, Singlet, Doublet, Doublet, Multiplet, Multiplet, Multiplet, Multiplet, Multiplet, Multiplet, Multiplet, Multiplet, Multiplet, Multiplet, Multiplet, Multiplet	0.151 (0.000 / 20)	0.146 (0.042 / 90)	- (- / 0)	- (- / 0)	0.091 (0.036 / 90)
**Choline**	4.1, 3.5, 3.2	Multiplet, Multiplet, Singlet	- (- / 0)	0.019 (0.005 / 30)	- (- / 0)	0.024 (0.006 / 40)	0.007 (0.005 / 30)
**Phenyllactic acid**	7.4, 7.3, 4.3, 3.1, 3.1, 2.9	Multiplet, Multiplet, Quartet, Doublet, Doublet, Quartet	- (- / 0)	0.022 (0.000 / 10)	0.080 (0.000 / 10)	0.051 (0.000 / 20)	- (- / 0)
**Inosinic acid**	8.6, 8.2, 6.1, 4.5, 4.4, 4.0	Singlet, Singlet, Doublet, Triplet, Multiplet, Multiplet	- (- / 0)	0.004 (0.000 / 10)	- (- / 0)	0.005 (0.000 / 20)	0.027 (0.023 / 20)
**Methylglutaric acid**	2.2, 2.0, 0.9	Multiplet, Multiplet, Doublet	- (- / 0)	0.140 (0.000 / 10)	0.305 (0.000 / 10)	- (- / 0)	0.008 (0.000 / 10)
**Tyrosine**	7.2, 6.9, 3.9, 3.2, 3.0	Quartet, Quartet, Quartet, Quartet, Quartet	- (- / 0)	0.328 (0.103 / 20)	- (- / 0)	0.843 (0.000 / 20)	0.358 (0.151 / 20)
**3,4-Dihydroxyhydrocinnamic acid**	6.9, 6.8, 6.7, 3.9, 3.2, 3.1, 3.0	Doublet, Doublet, Quartet, Quartet, Doublet, Doublet, Quartet	0.392 (0.000 / 20)	- (- / 0)	- (- / 0)	0.141 (0.000 / 20)	0.098 (0.018 / 30)
**5-Hydroxylysine**	3.9, 3.8, 3.2, 2.9, 2.0, 1.9, 1.7, 1.6	Multiplet, Multiplet, Multiplet, Multiplet, Multiplet, Multiplet, Multiplet, Multiplet	0.119 (0.028 / 40)	0.032 (0.011 / 60)	0.075 (0.051 / 80)	- (- / 0)	- (- / 0)
**myo-Inositol**	4.1, 3.6, 3.5, 3.3	Triplet, Triplet, Quartet, Triplet	0.200 (0.006 / 40)	- (- / 0)	- (- / 0)	0.077 (0.000 / 20)	0.027 (0.004 / 20)
**Gentisic acid**	7.3, 7.0, 6.8	Doublet, Quartet, Doublet	- (- / 0)	0.014 (0.000 / 10)	0.011 (0.000 / 10)	- (- / 0)	0.019 (0.007 / 20)
**N-Acetyl-L-phenylalanine**	7.9, 7.4, 7.3, 3.2, 2.9, 1.9	Doublet, Multiplet, Multiplet, Quartet, Quartet, Singlet	- (- / 0)	0.026 (0.000 / 10)	0.054 (0.010 / 30)	- (- / 0)	0.022 (0.006 / 20)
**Ribitol**	3.8, 3.7, 3.7	Multiplet, Doublet, Multiplet	0.550 (0.001 / 40)	- (- / 0)	0.367 (0.050 / 40)	0.307 (0.000 / 20)	- (- / 0)
**4-Aminohippuric acid**	7.7, 6.9, 3.9	Doublet, Doublet, Doublet	- (- / 0)	0.007 (0.000 / 10)	0.012 (0.005 / 20)	- (- / 0)	0.004 (0.000 / 10)
**Cysteine**	3.9, 3.1, 3.0	Quartet, Quartet, Quartet	0.086 (0.000 / 20)	0.174 (0.000 / 10)	0.323 (0.000 / 10)	- (- / 0)	- (- / 0)

**Acetoacetic acid**	3.4, 2.3	Singlet, Singlet	0.332 (0.000 / 20)	- (- / 0)	0.154 (0.000 / 10)	0.115 (0.048 / 100)	0.032 (0.007 / 30)
**1-Methyladenine**	8.4, 8.3, 3.9	Singlet, Singlet, Singlet	- (- / 0)	0.010 (0.002 / 30)	- (- / 0)	0.012 (0.002 / 80)	0.010 (0.005 / 20)
**Dopamine**	6.9, 6.8, 6.7, 3.2, 2.9	Doublet, Doublet, Quartet, Triplet, Triplet	0.098 (0.082 / 40)	0.021 (0.007 / 50)	- (- / 0)	0.082 (0.000 / 20)	0.016 (0.003 / 30)
**Pyridoxamine**	7.6, 4.3, 2.5	Singlet, Singlet, Singlet	0.220 (0.000 / 20)	- (- / 0)	- (- / 0)	0.068 (0.023 / 40)	0.065 (0.042 / 60)
**Urea**	5.8	Singlet	- (- / 0)	0.147 (0.020 / 30)	0.237 (0.010 / 20)	- (- / 0)	0.423 (0.079 / 30)
**4-Guanidinobuta-noic acid**	3.2, 2.2, 1.8	Triplet, Triplet, Multiplet	- (- / 0)	0.315 (0.000 / 10)	- (- / 0)	- (- / 0)	0.041 (0.007 / 20)
**Lysine**	3.8, 3.0, 1.9, 1.9, 1.7, 1.5, 1.4	Triplet, Triplet, Multiplet, Multiplet, Multiplet, Mul-tiplet, Multiplet	- (- / 0)	0.024 (0.006 / 40)	- (- / 0)	- (- / 0)	0.045 (0.036 / 90)
**N-Acetylputres-cine**	3.2, 3.0, 2.0, 1.7, 1.6	Triplet, Triplet, Singlet, Multiplet, Multiplet	0.009 (0.000 / 20)	- (- / 0)	- (- / 0)	- (- / 0)	0.019 (0.018 / 40)
**Mannitol**	3.9, 3.8, 3.8, 3.7.	Quartet, Doublet, Multi-plet, Quartet	- (- / 0)	0.294 (0.081 / 40)	- (- / 0)	- (- / 0)	0.350 (0.000 / 10)
**Acetylcysteine**	4.4, 2.9, 2.1	Multiplet, Multiplet, Sin-glet	- (- / 0)	- (- / 0)	- (- / 0)	0.073 (0.015 / 40)	0.043 (0.013 / 60)
**Ethanolamine**	3.8, 3.1	Triplet, Triplet	- (- / 0)	- (- / 0)	- (- / 0)	0.027 (0.019 / 60)	0.008 (0.000 / 10)
**Creatine**	3.9, 3.0	Singlet, Singlet	- (- / 0)	0.012 (0.002 / 60)	- (- / 0)	- (- / 0)	0.008 (0.003 / 70)
**1,11-Undecanedi-carboxylic acid**	2.2, 1.5, 1.3	Triplet, Multiplet, Singlet	0.307 (0.127 / 60)	- (- / 0)	- (- / 0)	- (- / 0)	0.064 (0.000 / 10)
**Vanillylmandelic acid**	7.1, 6.9, 3.9	Doublet, Multiplet, Singlet	- (- / 0)	- (- / 0)	- (- / 0)	0.091 (0.016 / 80)	0.039 (0.023 / 70)
**Asparagine**	4.0, 3.0, 2.9, 2.8	Quartet, Doublet, Doublet, Quartet	- (- / 0)	- (- / 0)	0.105 (0.000 / 10)	- (- / 0)	0.031 (0.000 / 10)
**Galactonic acid**	4.2, 4.0, 3.7, 3.6	Doublet, Multiplet, Triplet, Quartet	- (- / 0)	0.350 (0.157 / 20)	- (- / 0)	1.209 (0.000 / 20)	0.673 (0.091 / 20)
**Allothreonine**	4.4, 3.8, 1.2	Multiplet, Doublet, Dou-blet	- (- / 0)	0.142 (0.000 / 10)	- (- / 0)	- (- / 0)	0.049 (0.002 / 20)
**Glycocholic acid**	4.0, 3.9, 3.5, 2.4, 2.2, 2.1, 2.0, 1.9, 1.7, 1.6, 1.3, 1.1, 1.0, 0.9, 0.7	Singlet, Singlet, Multiplet, Multiplet, Multiplet, Mul-tiplet, Singlet, Multiplet, Multiplet, Multiplet, Mul-tiplet, Multiplet, Multiplet, Singlet, Singlet	- (- / 0)	0.009 (0.006 / 30)	0.014 (0.010 / 40)	- (- / 0)	- (- / 0)
**Octanal**	9.7, 2.5, 0.9	Triplet, Multiplet, Triplet	- (- / 0)	1.623 (0.000 / 10)	- (- / 0)	- (- / 0)	1.602 (0.000 / 10)
**Leucinic acid**	4.3, 1.8, 1.6, 0.9	Quartet, Multiplet, Multi-plet, Quartet	- (- / 0)	- (- / 0)	0.172 (0.000 / 10)	- (- / 0)	0.018 (0.000 / 10)
**Hippuric acid**	7.8, 7.6, 7.5, 4.0	Multiplet, Multiplet, Mul-tiplet, Doublet	0.019 (0.000 / 20)	- (- / 0)	0.014 (0.000 / 10)	- (- / 0)	- (- / 0)
**Isopropyl alcohol**	4.0, 1.2	Multiplet, Doublet	- (- / 0)	0.017 (0.000 / 10)	0.029 (0.000 / 10)	- (- / 0)	- (- / 0)
**3-Hydroxybutyric acid**	4.2, 2.4, 2.3, 1.2	Doublet, Quartet, Quartet, Doublet	- (- / 0)	- (- / 0)	0.795 (0.000 / 10)	- (- / 0)	0.416 (0.000 / 10)
**Undecanoic acid**	2.1, 1.5, 1.3, 0.8	Triplet, Triplet, Multiplet, Triplet	- (- / 0)	0.057 (0.000 / 10)	- (- / 0)	- (- / 0)	0.384 (0.000 / 10)
**2-Piperidinone**	3.3, 2.3, 1.8	Multiplet, Triplet, Multi-plet	- (- / 0)	0.043 (0.000 / 10)	0.060 (0.000 / 10)	- (- / 0)	- (- / 0)
**Agmatine**	3.2, 3.0, 1.7	Triplet, Triplet, Multiplet	- (- / 0)	- (- / 0)	- (- / 0)	0.382 (0.000 / 20)	0.245 (0.000 / 10)
**2-Furoic acid**	7.6, 7.0, 6.5	Quartet, Quartet, Quartet	0.051 (0.000 / 20)	- (- / 0)	- (- / 0)	- (- / 0)	0.009 (0.000 / 20)
**Deoxyguanosine**	8.0, 6.3, 4.6, 4.1, 3.8, 3.8, 2.8, 2.5	Singlet, Triplet, Multiplet, Quartet, Quartet, Quartet, Multiplet, Multiplet	- (- / 0)	0.025 (0.003 / 30)	- (- / 0)	- (- / 0)	0.026 (0.003 / 40)

**Alloisoleucine**	3.7, 2.1, 1.4, 1.3, 0.9	Doublet, Multiplet, Multiplet, Multiplet, Multiplet	0.215 (0.000 / 20)	0.063 (0.000 / 10)	- (- / 0)	0.226 (0.000 / 20)	0.005 (0.000 / 10)
**D-Alpha-amino-butyric acid**	3.7, 1.9, 1.0	Triplet, Multiplet, Triplet	- (- / 0)	- (- / 0)	0.176 (0.141 / 20)	- (- / 0)	0.054 (0.000 / 10)
**Oxoglutaric acid**	3.0, 2.4	Triplet, Triplet	- (- / 0)	- (- / 0)	0.215 (0.000 / 10)	0.197 (0.000 / 20)	0.092 (0.000 / 10)
**Protocatechuic acid**	7.4, 7.4, 6.9	Doublet, Quartet, Doublet	- (- / 0)	0.016 (0.000 / 10)	- (- / 0)	0.014 (0.002 / 40)	- (- / 0)
**Butanal**	9.7, 2.5, 1.6, 0.9	Triplet, Multiplet, Multiplet, Multiplet	- (- / 0)	0.614 (0.000 / 10)	- (- / 0)	0.525 (0.000 / 20)	- (- / 0)
**Dihydrouracil**	3.4, 2.7	Triplet, Triplet	- (- / 0)	0.066 (0.000 / 10)	- (- / 0)	- (- / 0)	0.003 (0.000 / 10)
**6-Hydroxynico-tinic acid**	8.1, 6.6	Multiplet, Quartet	- (- / 0)	- (- / 0)	0.064 (0.000 / 10)	- (- / 0)	0.039 (0.000 / 10)
**Taurine**	3.4, 3.3	Triplet, Triplet	- (- / 0)	- (- / 0)	- (- / 0)	0.039 (0.000 / 20)	0.006 (0.000 / 10)
**Saccharopine**	3.7, 3.6, 3.0, 2.4, 2.1, 1.9, 1.8, 1.5	Quartet, Triplet, Multiplet, Multiplet, Multiplet, Multiplet, Multiplet, Multiplet	- (- / 0)	- (- / 0)	- (- / 0)	0.728 (0.000 / 20)	0.481 (0.371 / 20)
**Citrulline**	3.8, 3.1, 1.9, 1.6	Triplet, Multiplet, Multiplet, Multiplet	- (- / 0)	- (- / 0)	- (- / 0)	0.046 (0.000 / 20)	0.061 (0.000 / 10)
**Maleic acid**	6.1	Singlet	- (- / 0)	0.001 (0.000 / 10)	- (- / 0)	- (- / 0)	0.439 (0.000 / 10)
**O-Phosphoethan-olamine**	4.0, 3.2	Multiplet, Triplet	- (- / 0)	- (- / 0)	- (- / 0)	0.108 (0.000 / 20)	0.015 (0.000 / 10)
**2-Hydroxycaproic acid**	4.0, 1.7, 1.6, 1.3, 0.9	Quartet, Multiplet, Multiplet, Multiplet, Triplet	- (- / 0)	- (- / 0)	0.258 (0.000 / 10)	- (- / 0)	0.031 (0.000 / 10)
**Glycolic acid**	3.9	Singlet	- (- / 0)	0.007 (0.002 / 100)	- (- / 0)	- (- / 0)	0.011 (0.003 / 90)
**Gamma-Glu-tamylcysteine**	4.6, 3.9, 3.0, 2.5, 2.2	Multiplet, Triplet, Multiplet, Multiplet, Multiplet	- (- / 0)	0.026 (0.000 / 20)	- (- / 0)	- (- / 0)	0.006 (0.000 / 10)
**Dimethylamine**	2.5	Singlet	- (- / 0)	- (- / 0)	- (- / 0)	0.012 (0.003 / 80)	0.004 (0.000 / 10)
**Guaiacol**	6.9, 6.9, 3.9	Multiplet, Multiplet, Singlet	- (- / 0)	0.062 (0.024 / 40)	- (- / 0)	- (- / 0)	0.064 (0.026 / 50)
**Ethyl isopropyl ketone**	2.8, 2.6, 1.0, 1.0	Multiplet, Quartet, Doublet, Triplet	- (- / 0)	0.733 (0.204 / 60)	1.546 (0.890 / 70)	- (- / 0)	- (- / 0)
**Delta-Hexanolac-tone**	4.6, 3.8, 2.6, 2.5, 2.2, 2.0, 1.9, 1.5, 1.3, 1.2	Multiplet, Multiplet, Multiplet, Multiplet, Triplet, Multiplet, Multiplet, Multiplet, Doublet, Doublet	- (- / 0)	0.236 (0.207 / 20)	1.011 (0.000 / 10)	- (- / 0)	- (- / 0)
**(S)-3-Hydroxyiso-butyric acid**	3.7, 2.6, 1.1	Multiplet, Multiplet, Doublet	- (- / 0)	- (- / 0)	0.034 (0.005 / 20)	0.055 (0.012 / 40)	- (- / 0)
**Citraconic acid**	5.6, 2.0	Doublet, Doublet	- (- / 0)	- (- / 0)	- (- / 0)	0.107 (0.000 / 20)	0.033 (0.011 / 50)
**Fructose**	4.1, 4.1, 4.0, 4.0, 3.9, 3.8, 3.8, 3.8, 3.7, 3.7, 3.7, 3.6, 3.6, 3.5	Quartet, Quartet, Quartet, Multiplet, Quartet, Multiplet, Quartet, Doublet, Doublet, Quartet, Quartet, Doublet, Doublet, Doublet	- (- / 0)	3.931 (0.512 / 20)	- (- / 0)	- (- / 0)	2.705 (1.244 / 60)
**Fucose**	5.2, 4.6, 4.2, 3.9, 3.8, 3.6, 3.5, 1.3, 1.2	Doublet, Doublet, Quartet, Quartet, Multiplet, Quartet, Quartet, Doublet, Doublet	0.214 (0.000 / 20)	0.029 (0.025 / 40)	0.034 (0.020 / 20)	- (- / 0)	- (- / 0)
**Dihydrothymine**	3.5, 3.2, 3.2, 3.2, 2.8, 1.2	Quartet, Singlet, Doublet, Singlet, Multiplet, Doublet	- (- / 0)	0.146 (0.000 / 10)	- (- / 0)	- (- / 0)	0.030 (0.003 / 20)
**o-Tyrosine**	7.2, 6.9, 4.0, 3.3, 3.3, 3.0	Multiplet, Multiplet, Quartet, Doublet, Doublet, Quartet	- (- / 0)	0.114 (0.051 / 30)	- (- / 0)	- (- / 0)	0.082 (0.059 / 60)
**Arabitol**	3.9, 3.8, 3.7, 3.7, 3.6	Multiplet, Quartet, Multiplet, Multiplet, Quartet	- (- / 0)	0.342 (0.000 / 10)	- (- / 0)	- (- / 0)	0.154 (0.000 / 10)

**Benzoic acid**	7.9, 7.5, 7.5	Multiplet, Multiplet, Triplet	- (- / 0)	- (- / 0)	0.028 (0.000 / 10)	- (- / 0)	0.007 (0.000 / 10)
**Aminoadipic acid**	3.9, 3.7, 2.3, 2.2, 2.1, 1.8, 1.6	Multiplet, Quartet, Multiplet, Multiplet, Multiplet, Multiplet, Multiplet	0.224 (0.000 / 20)	- (- / 0)	- (- / 0)	- (- / 0)	- (- / 0)
**Octanoylcarnitine**	5.6, 3.9, 3.6, 3.6, 3.2, 2.7, 2.7, 2.6, 2.4, 1.6, 1.3, 0.8	Quartet, Quartet, Singlet, Singlet, Singlet, Doublet, Doublet, Quartet, Multiplet, Triplet, Multiplet, Triplet	0.802 (0.000 / 20)	- (- / 0)	- (- / 0)	- (- / 0)	- (- / 0)
**Hexanoylcarnitine**	5.6, 3.9, 3.6, 3.6, 3.2, 2.6, 2.6, 2.5, 1.6, 1.3, 0.9	Quartet, Quartet, Singlet, Singlet, Singlet, Doublet, Doublet, Multiplet, Multiplet, Multiplet, Triplet	- (- / 0)	0.001 (0.000 / 10)	- (- / 0)	0.049 (0.000 / 20)	- (- / 0)
**Homocitrulline**	6.3, 3.7, 3.1, 1.9, 1.5, 1.4, 1.4	Singlet, Quartet, Quartet, Multiplet, Multiplet, Multiplet, Multiplet,	- (- / 0)	0.018 (0.002 / 20)	- (- / 0)	- (- / 0)	- (- / 0)
**1-Hexadecanol**	3.5, 1.5, 1.3, 0.9	Triplet, Multiplet, Multiplet, Triplet	- (- / 0)	0.062 (0.000 / 10)	- (- / 0)	- (- / 0)	- (- / 0)
**Azelaic acid**	2.2, 1.5, 1.3	Triplet, Multiplet, Multiplet	- (- / 0)	0.147 (0.000 / 10)	- (- / 0)	- (- / 0)	- (- / 0)
**Gamma-Aminobutyric acid**	3.0, 2.3, 1.9	Triplet, Triplet, Multiplet	- (- / 0)	- (- / 0)	0.161 (0.000 / 10)	- (- / 0)	- (- / 0)
**5-Aminopentanoic acid**	3.0, 2.2, 1.6	Triplet, Triplet, Multiplet	- (- / 0)	- (- / 0)	0.196 (0.033 / 20)	- (- / 0)	- (- / 0)
**Ethylmalonic acid**	3.0, 1.7, 0.9	Triplet, Multiplet, Triplet	- (- / 0)	- (- / 0)	0.202 (0.030 / 20)	- (- / 0)	- (- / 0)
**Ketoleucine**	2.7, 2.1, 0.9	Doublet, Multiplet, Doublet	- (- / 0)	- (- / 0)	0.519 (0.000 / 10)	- (- / 0)	- (- / 0)
**2-Hydroxyadipic acid**	4.1, 2.4, 1.7	Quartet, Multiplet, Multiplet	- (- / 0)	- (- / 0)	0.248 (0.000 / 10)	- (- / 0)	- (- / 0)
**3-Hexanone**	2.5, 1.6, 1.0, 0.9	Multiplet, Multiplet, Quartet, Triplet	- (- / 0)	- (- / 0)	1.178 (0.000 / 10)	- (- / 0)	- (- / 0)
**Glyceric acid**	4.1, 3.8, 3.7	Quartet, Quartet, Quartet	- (- / 0)	- (- / 0)	0.099 (0.000 / 10)	- (- / 0)	- (- / 0)
**Iditol**	3.8, 3.7, 3.6	Multiplet, Multiplet, Quartet	- (- / 0)	- (- / 0)	- (- / 0)	1.309 (0.000 / 20)	- (- / 0)
**Xylitol**	3.8, 3.8, 3.7, 3.7, 3.7, 3.6, 3.6	Multiplet, Multiplet, Doublet, Doublet, Doublet, Triplet, Doublet	- (- / 0)	- (- / 0)	- (- / 0)	0.006 (0.000 / 20)	- (- / 0)
**Levulinic acid**	2.8, 2.4, 2.2	Triplet, Triplet, Singlet	- (- / 0)	- (- / 0)	- (- / 0)	1.590 (0.000 / 20)	- (- / 0)
**Acetamide**	7.5, 6.8, 2.0	Singlet, Singlet, Singlet	- (- / 0)	- (- / 0)	- (- / 0)	0.655 (0.000 / 20)	- (- / 0)
**Glucaric acid**	4.1, 4.1, 4.1, 3.9	Doublet, Doublet, Quartet, Triplet	- (- / 0)	- (- / 0)	- (- / 0)	0.042 (0.000 / 20)	- (- / 0)
**Guanine**	7.7	Singlet	- (- / 0)	- (- / 0)	- (- / 0)	0.074 (0.000 / 20)	0.014 (0.002 / 40)
**Dihydroxyacetone**	4.4	Singlet	- (- / 0)	- (- / 0)	- (- / 0)	- (- / 0)	0.020 (0.005 / 60)
**Diethanolamine**	3.9, 3.2	Triplet, Quartet	- (- / 0)	- (- / 0)	- (- / 0)	- (- / 0)	0.131 (0.088 / 20)
**Serine**	4.0, 3.9, 3.8	Quartet, Quartet, Quartet	- (- / 0)	- (- / 0)	- (- / 0)	- (- / 0)	0.009 (0.003 / 30)
**Propylene glycol**	3.9, 3.5, 3.4, 1.1	Multiplet, Quartet, Quartet, Doublet	- (- / 0)	- (- / 0)	- (- / 0)	- (- / 0)	0.008 (0.003 / 40)
**Cyclohexanone**	2.4, 1.9, 1.7	Triplet, Multiplet, Multiplet	- (- / 0)	- (- / 0)	- (- / 0)	- (- / 0)	0.084 (0.000 / 10)
**Diaminopimelic acid**	3.8, 3.8, 1.9, 1.5	Triplet, Quartet, Multiplet, Multiplet	- (- / 0)	- (- / 0)	- (- / 0)	- (- / 0)	0.045 (0.000 / 10)
**Homocysteine**	3.9, 2.7, 2.1	Quartet, Multiplet, Multiplet	- (- / 0)	- (- / 0)	- (- / 0)	- (- / 0)	0.083 (0.000 / 10)

**Homoserine**	3.9, 3.8, 2.1, 2.0	Quartet, Multiplet, Multiplet, Multiplet	- (- / 0)	- (- / 0)	- (- / 0)	- (- / 0)	0.021 (0.000 / 10)
**Heptanoic acid**	2.2, 1.5, 1.3, 0.9	Triplet, Multiplet, Multiplet, Triplet	- (- / 0)	- (- / 0)	- (- / 0)	- (- / 0)	0.187 (0.070 / 50)
**Gamma-Caprolactone**	4.7, 2.6, 2.4, 1.9, 1.8, 1.7, 0.9	Multiplet, Multiplet, Multiplet, Multiplet, Multiplet, Multiplet, Triplet	- (- / 0)	- (- / 0)	- (- / 0)	- (- / 0)	0.041 (0.000 / 10)
**Benzaldehyde**	9.9, 8.0, 7.8, 7.6	Singlet, Quartet, Singlet, Triplet	- (- / 0)	- (- / 0)	- (- / 0)	- (- / 0)	0.128 (0.000 / 10)
**2,3-Butanediol**	3.7, 3.6, 1.1	Multiplet, Multiplet, Multiplet	- (- / 0)	- (- / 0)	- (- / 0)	- (- / 0)	0.035 (0.000 / 10)
**Isocitric acid**	4.0, 3.0, 2.5, 2.4	Doublet, Multiplet, Quartet, Quartet	- (- / 0)	- (- / 0)	- (- / 0)	- (- / 0)	0.030 (0.000 / 10)
**Spermidine**	2.6, 1.6, 1.5	Multiplet, Multiplet, Multiplet	- (- / 0)	- (- / 0)	- (- / 0)	- (- / 0)	0.049 (0.000 / 10)
**Cytosine**	7.5, 6.0	Doublet, Doublet	- (- / 0)	- (- / 0)	- (- / 0)	- (- / 0)	0.014 (0.000 / 10)
**Glycerol 3-phosphate**	3.8, 3.7, 3.6	Multiplet, Quartet, Quartet	- (- / 0)	- (- / 0)	- (- / 0)	- (- / 0)	0.039 (0.000 / 10)
**Pyroglutamic acid**	4.2, 2.5, 2.4, 2.0	Quartet, Multiplet, Multiplet, Multiplet	- (- / 0)	- (- / 0)	- (- / 0)	- (- / 0)	0.002 (0.000 / 10)
**Tetrahydrofuran**	3.7, 1.9	Multiplet, Multiplet	- (- / 0)	- (- / 0)	- (- / 0)	- (- / 0)	0.401 (0.000 / 10)
**Dimethylmalonic acid**	1.4	Singlet	- (- / 0)	- (- / 0)	- (- / 0)	- (- / 0)	0.223 (0.000 / 10)

Table S2. Average concentrations (mM) of select metabolites in rat feces that were processed by ultrafiltration, no extraction without baseline correction, Bligh-Dyer (BD) extraction without baseline correction, no extraction with baseline correction, and BD with baseline correction. Metabolites were identified and quantified using Chenomx, and only metabolites with a percent occurrence of 50 or higher are shown. Metabolite numbers correspond to Figure 4, and the numbers presented in brackets correspond to the standard deviation and percent occurrence of each metabolite, respectively.

#	Compound Name	No Extraction	Bligh-Dyer	Ultra-filtration	No Extraction BC	Bligh-Dyer BC
1	1,3-Dimethyluric acid	0.078 (0.041/100)	0.039 (0.011/80)	0.080 (0.033/100)	0.058 (0.029/100)	0.036 (0.014/80)
2	1-Methyladenosine	1.837 (0.645/75)	0.445 (0.117/70)	1.171 (0.639/80)	1.013 (0.462/75)	0.350 (0.139/80)
3	2-Isopropylmalic acid	0.058 (0.025/50)	0.019 (0.005/60)	0.049 (0.020/90)	0.026 (0.013/100)	0.012 (0.003/100)
4	3-Hydroxyisovaleric acid	0.101 (0.034/50)	0.033 (0.012/90)	0.138 (0.061/80)	0.070 (0.034/75)	0.031 (0.012/90)
5	3-Hydroxymandelic acid	0.037 (0.010/50)	0.013 (0.006/80)	0.035 (0.006/90)	0.031 (0.003/100)	0.029 (0.058/70)
6	3-Hydroxymethylglutaric acid	0.112 (0.010/75)	0.036 (0.012/90)	0.101 (0.019/90)	0.063 (0.014/100)	0.032 (0.008/90)
7	3-Hydroxyphenylacetic acid	2.543 (0.247/100)	1.005 (0.177/70)	1.793 (0.436/90)	2.544 (0.246/50)	1.039 (0.159/80)
8	3-Methylxanthine	0.042 (0.010/75)	0.009 (0.004/80)	0.022 (0.009/70)	0.008 (0.005/100)	0.002 (0.001/80)
9	4-Pyridoxic acid	0.054 (0.030/75)	0.028 (0.009/100)	0.055 (0.023/100)	0.042 (0.031/100)	0.020 (0.014/90)
10	5-Hydroxyindoleacetic acid	0.115 (0.044/50)	0.030 (0.009/80)	0.113 (0.046/90)	0.110 (0.056/75)	0.033 (0.008/90)
11	7-Methylxanthine	11.815 (1.319/75)	4.602 (0.774/90)	8.367 (1.864/90)	11.802 (1.312/100)	4.652 (0.798/90)
12	Acetic acid	0.521 (0.445/100)	0.194 (0.100/100)	0.565 (0.306/100)	0.531 (0.433/100)	0.189 (0.094/90)
13	Allantoin	0.010 (0.002/100)	0.004 (0.001/60)	0.007 (0.002/100)	0.006 (0.002/100)	0.008 (0.011/60)
14	Alpha-D-Glucose	0.752 (0.425/75)	0.200 (0.109/50)	0.350 (0.180/90)	0.079 (0.038/75)	0.126 (0.070/70)
15	Argininosuccinic acid	1.147 (0.310/50)	0.167 (0.057/70)	1.000 (0.251/60)	1.381 (0.319/100)	0.300 (0.043/90)
16	Biotin	0.186 (0.147/100)	0.050 (0.035/100)	0.085 (0.061/100)	0.188 (0.171/50)	0.079 (0.028/90)
17	Butyric acid	0.037 (0.015/100)	0.011 (0.004/100)	0.034 (0.011/100)	0.049 (0.013/100)	0.038 (0.013/90)
18	Cytidine monophosphate	1.129 (0.188/75)	0.333 (0.106/50)	0.887 (0.210/80)	0.117 (0.053/50)	0.112 (0.059/60)
19	D-Glucose	0.080 (0.043/50)	0.015 (0.010/90)	0.046 (0.017/100)	0.014 (0.002/100)	0.010 (0.008/60)
20	D-Maltose	0.028 (0.017/75)	0.004 (0.003/50)	0.011 (0.005/80)	0.004 (0.002/50)	0.003 (0.001/70)
21	Ethanol	0.634 (0.122/75)	0.121 (0.055/90)	0.239 (0.262/100)	0.378 (0.231/75)	0.285 (0.211/90)
22	Formic acid	0.751 (0.254/50)	0.132 (0.078/80)	0.413 (0.166/100)	0.142 (0.074/75)	0.442 (1.226/70)
23	Galacturonic acid	0.147 (0.051/100)	0.026 (0.014/100)	0.086 (0.013/100)	0.022 (0.001/100)	0.131 (0.313/90)
24	Glucose 6-phosphate	0.747 (0.137/75)	0.236 (0.078/80)	1.155 (0.971/100)	0.922 (0.775/75)	0.227 (0.115/50)
25	Glycine	3.848 (1.948/50)	1.324 (0.467/90)	2.551 (1.217/90)	3.734 (2.067/75)	1.305 (0.457/90)

26	Homoveratric acid	0.033 (0.005/100)	0.007 (0.001/50)	0.022 (0.012/80)	0.016 (0.007/50)	0.007 (0.001/100)
27	Hydrocinnamic acid	0.166 (0.027/100)	0.068 (0.024/90)	0.094 (0.058/90)	0.132 (0.022/100)	0.035 (0.023/90)
28	Hydroxyacetone	5.395 (1.241/100)	1.411 (0.457/90)	5.416 (0.907/80)	2.046 (1.035/75)	0.691 (0.397/90)
29	Isovalerylglycine	0.036 (0.018/50)	0.004 (0.002/60)	0.012 (0.013/80)	0.004 (0.001/75)	0.004 (0.002/60)
30	L-Alanine	0.320 (0.116/75)	0.045 (0.014/100)	0.213 (0.069/100)	0.108 (0.067/100)	0.048 (0.012/90)
31	L-Arabinose	0.448 (0.015/100)	0.086 (0.033/50)	0.372 (0.077/90)	0.334 (0.058/50)	0.097 (0.047/50)
32	L-Phenylalanine	6.985 (2.362/100)	0.460 (0.288/70)	0.576 (0.638/100)	2.502 (0.997/100)	0.719 (0.405/70)
33	L-Valine	0.325 (0.184/100)	0.124 (0.040/100)	0.176 (0.126/90)	0.279 (0.140/100)	0.153 (0.065/100)
34	Malic acid	0.179 (0.043/100)	0.019 (0.008/100)	0.124 (0.056/100)	0.159 (0.047/100)	0.023 (0.013/100)
35	Methyl isobutyl ketone	0.072 (0.059/100)	0.074 (0.019/70)	0.156 (0.041/100)	0.157 (0.050/100)	0.038 (0.026/50)
36	Methylimidazoleacetic acid	0.214 (0.115/100)	0.154 (0.076/100)	0.199 (0.083/100)	0.211 (0.117/100)	0.165 (0.072/90)
37	N-Acetyl-L-methionine	0.441 (0.090/100)	0.113 (0.058/70)	0.329 (0.132/60)	0.227 (0.062/100)	0.074 (0.030/100)
38	N-Acetylneuraminic acid	0.106 (0.069/100)	0.073 (0.039/100)	0.091 (0.053/100)	0.076 (0.038/100)	0.062 (0.019/100)
39	N-Formyl-L-methionine	1.099 (0.181/75)	0.343 (0.141/50)	0.979 (0.191/100)	0.351 (0.189/100)	0.236 (0.063/70)
40	Nicotinic acid	0.181 (0.053/100)	0.038 (0.010/80)	0.118 (0.041/100)	0.116 (0.011/100)	0.035 (0.009/70)
41	Pantothenic acid	0.091 (0.016/100)	0.035 (0.011/100)	0.074 (0.013/100)	0.076 (0.017/100)	0.037 (0.010/100)
42	Phenylacetic acid	0.099 (0.030/50)	0.024 (0.010/80)	0.045 (0.021/80)	0.053 (0.001/75)	0.024 (0.007/90)
43	Propionic acid	0.158 (0.077/100)	0.015 (0.005/100)	0.058 (0.037/100)	0.020 (0.003/100)	0.037 (0.022/90)
44	Raffinose	0.338 (0.152/75)	0.159 (0.054/80)	0.477 (0.166/70)	0.340 (0.134/100)	0.168 (0.052/90)
45	Riboflavin	0.220 (0.064/100)	0.050 (0.011/100)	0.164 (0.028/100)	0.663 (0.132/100)	0.147 (0.053/90)
46	Sebacic acid	0.028 (0.001/100)	0.088 (0.027/90)	0.034 (0.017/100)	0.026 (0.004/75)	0.091 (0.026/80)
47	Succinic acid	0.264 (0.166/100)	0.094 (0.030/100)	0.163 (0.128/100)	0.081 (0.016/100)	0.054 (0.008/90)
48	Sumiki's acid	0.399 (0.157/75)	0.160 (0.027/80)	0.331 (0.090/80)	0.402 (0.153/50)	0.164 (0.026/50)
49	Thiamine	0.151 (0.083/75)	0.034 (0.018/70)	0.094 (0.030/70)	0.173 (0.058/75)	0.060 (0.021/60)
50	Undecanedioic acid	4.792 (2.549/100)	1.785 (1.351/100)	4.081 (1.655/100)	0.396 (0.097/100)	0.357 (0.243/80)
51	Urocanic acid	0.016 (0.003/100)	0.012 (0.002/70)	0.021 (0.004/90)	0.022 (0.008/75)	0.014 (0.004/60)
52	Xanthine	0.121 (0.107/75)	0.057 (0.030/100)	0.197 (0.155/100)	0.330 (0.073/100)	0.065 (0.014/90)

Table S3. Average concentrations (mM) of all metabolites identified in chicken feces that were processed by ultrafiltration (UF), no extraction without baseline correction, Bligh-Dyer (BD) extraction without baseline correction, no extraction with baseline correction (BC), and BD with BC. Metabolites were identified and quantified using Chenomx. Metabolite numbers correspond to Supplemental Figure S4, and the numbers presented in brackets correspond to the standard deviation and percent occurrence of each metabolite, respectively.

#	Compound Name	Chemical shift (ppm)	Multiplicity	No Extraction av mM (STD / % occur.)	BD av mM (STD / % occur.)	UF av mM (STD / % occur.)	No Extraction BC av mM (STD % oc-cur.)	BD BC av mM (STD / % occur.)
1	2-Hydroxy-butyrate	4.0, 1.7, 1.6, 0.9	Quartet, Multiplet, Mul-tiplet, Triplet	0.121 (0.051 / 75)	0.051 (0.022 / 75)	0.059 (0.028 / 75)	- (- / 0)	0.037 (0.007 / 50)
2	Acetate	1.9	Singlet	2.046 (0.647 / 100)	1.124 (0.492 / 100)	0.864 (0.322 / 87.5)	2.045 (0.648 / 100)	1.124 (0.491 / 100)
3	Alanine	3.8, 1.5	Quartet, Doublet	0.134 (0.055 / 100)	0.070 (0.026 / 100)	0.059 (0.019 / 87.5)	0.134 (0.055 / 100)	0.070 (0.026 / 100)
4	Aspartate	3.9, 2.8, 2.7	Quartet, Doublet, Dou-blet	0.105 (0.050 / 50)	0.034 (0.012 / 50)	0.044 (0.011 / 87.5)	0.072 (0.023 / 62.5)	0.034 (0.012 / 50)
5	Betaine	3.9, 3.3	Singlet, Singlet	0.117 (0.047 / 87.5)	0.092 (0.058 / 100)	0.076 (0.056 / 87.5)	0.132 (0.120 / 100)	0.092 (0.058 / 100)
6	Butyrate	2.1, 1.5, 0.9	Triplet, Multiplet, Tri-plet	0.117 (0.123 / 50)	0.083 (0.095 / 87.5)	0.033 (0.015 / 37.5)	0.154 (0.090 / 100)	0.092 (0.087 / 100)
7	Choline	4.1, 3.5, 3.2	Multiplet, Multiplet, Singlet	0.140 (0.096 / 100)	0.127 (0.040 / 100)	0.076 (0.040 / 87.5)	0.139 (0.096 / 100)	0.127 (0.040 / 100)
8	Citrate	2.7, 2.5	Doublet, Doublet	0.017 (0.000 / 12.5)	- (- / 0)	- (- / 0)	0.021 (0.007 / 37.5)	0.008 (0.000 / 12.5)

9	Creatine	3.9, 3.0	Singlet, Singlet	0.097 (0.125 / 75)	0.129 (0.110 / 100)	0.067 (0.059 / 75)	0.109 (0.120 / 100)	0.130 (0.107 / 100)
10	Creatinine	4.0, 3.0	Singlet, Singlet	0.166 (0.070 / 100)	0.035 (0.016 / 100)	0.074 (0.037 / 87.5)	0.117 (0.073 / 100)	0.034 (0.023 / 100)
11	Dimethylamine	2.7	Singlet	0.066 (0.032 / 100)	0.009 (0.004 / 25)	0.043 (0.017 / 87.5)	0.054 (0.039 / 100)	0.007 (0.004 / 75)
12	Formate	8.4	Singlet	0.560 (0.187 / 100)	0.309 (0.115 / 100)	0.295 (0.081 / 87.5)	0.560 (0.187 / 100)	0.309 (0.115 / 100)
13	Galactose	5.3, 4.6, 4.1, 4.0, 3.9, 3.9, 3.8, 3.8, 3.7, 3.7, 3.7, 3.7, 3.6, 3.5	Doublet, Doublet, Triplet, Doublet, Doublet, Doublet, Doublet, Quartet, Doublet, Doublet, Doublet, Quartet, Doublet, Quartet	0.288 (0.221 / 37.5)	0.050 (0.028 / 50)	0.088 (0.034 / 87.5)	0.331 (0.000 / 12.5)	0.230 (0.043 / 50)
14	Glucose	5.2, 4.6, 3.9, 3.8, 3.8, 3.8, 3.7, 3.7, 3.5, 3.5, 3.5, 3.4, 3.4, 3.2	Doublet, Doublet, Doublet, Doublet, Multiplet, Doublet, Doublet, Triplet, Doublet, Triplet, Multiplet, Triplet, Triplet, Quartet	0.371 (0.176 / 87.5)	0.059 (0.040 / 87.5)	0.333 (0.136 / 87.5)	0.370 (0.117 / 62.5)	0.125 (0.067 / 62.5)
15	Glutamate	3.8, 2.4, 2.3, 2.1, 2.0	Quartet, Multiplet, Multiplet, Multiplet, Multiplet	- (- / 0)	0.160 (0.095 / 25)	- (- / 0)	- (- / 0)	0.146 (0.057 / 50)
16	Glycine	3.6	Singlet	0.080 (0.000 / 12.5)	0.050 (0.023 / 100)	0.112 (0.045 / 75)	0.080 (0.000 / 12.5)	0.050 (0.023 / 100)
17	Isoleucine	3.7, 2.0, 1.5, 1.2, 1.0, 0.9	Doublet, Multiplet, Multiplet, Multiplet, Doublet, Triplet	0.080 (0.033 / 100)	0.033 (0.013 / 100)	0.031 (0.011 / 87.5)	0.080 (0.033 / 100)	0.032 (0.011 / 75)
18	Lactate	4.1, 1.3	Quartet, Doublet	0.185 (0.000 / 12.5)	0.169 (0.050 / 62.5)	0.117 (0.000 / 12.5)	0.175 (0.000 / 12.5)	0.135 (0.061 / 100)
19	Lysine	3.8, 3.0, 1.9, 1.9, 1.7, 1.5, 1.4	Triplet, Triplet, Multiplet, Multiplet, Multiplet, Multiplet, Multiplet	0.191 (0.000 / 12.5)	0.032 (0.000 / 12.5)	0.074 (0.000 / 12.5)	0.114 (0.033 / 37.5)	0.049 (0.015 / 25)
20	Methionine	3.9, 2.6, 2.2, 2.1, 2.1	Quartet, Triplet, Multiplet, Singlet, Multiplet	0.026 (0.011 / 50)	0.009 (0.006 / 37.5)	0.014 (0.006 / 75)	0.015 (0.006 / 75)	0.008 (0.004 / 100)
21	Methylamine	2.6	Singlet	0.012 (0.000 / 12.5)	- (- / 0)	0.007 (0.002 / 62.5)	0.004 (0.004 / 100)	0.001 (0.000 / 12.5)
22	N-Acetylglucosamine	8.2, 8.1, 5.2, 4.7, 3.9, 3.9, 3.8, 3.8, 3.8, 3.8, 3.7, 3.7, 3.5, 3.5, 3.5, 3.4, 2.0, 2.0	Doublet, Doublet, Doublet, Doublet, Doublet, Multiplet, Multiplet, Doublet, Doublet, Multiplet, Multiplet, Quartet, Triplet, Triplet, Multiplet, Triplet, Singlet, Singlet	0.145 (0.066 / 100)	0.056 (0.012 / 62.5)	0.054 (0.027 / 87.5)	0.106 (0.047 / 100)	0.046 (0.014 / 75)
23	N-Acetyltyrosine	7.7, 7.2, 6.8, 4.4, 3.1, 2.8, 1.9	Doublet, Doublet, Doublet, Multiplet, Quartet, Quartet, Singlet	0.019 (0.006 / 37.5)	0.015 (0.005 / 87.5)	0.015 (0.002 / 25)	0.015 (0.007 / 100)	0.013 (0.005 / 87.5)
24	Phenylalanine	7.4, 7.4, 7.3, 4.0, 3.3, 3.1	Triplet, Triplet, Doublet, Quartet, Quartet, Quartet	0.084 (0.026 / 100)	0.040 (0.012 / 62.5)	0.039 (0.009 / 87.5)	0.079 (0.026 / 100)	0.040 (0.012 / 62.5)
25	Propionate	2.2, 1.0	Quartet, Triplet	0.071 (0.000 / 12.5)	0.030 (0.017 / 25)	0.024 (0.000 / 12.5)	0.037 (0.025 / 50)	0.017 (0.007 / 50)

26	Succinate	2.4	Singlet	0.020 (0.009 / 87.5)	0.013 (0.005 / 100)	0.008 (0.002 / 87.5)	0.018 (0.009 / 100)	0.013 (0.005 / 100)
27	Threonine	4.2, 3.6, 1.3	Multiplet, Doublet, Doublet	0.226 (0.120 / 75)	0.067 (0.043 / 100)	0.106 (0.063 / 87.5)	0.218 (0.119 / 75)	0.044 (0.035 / 100)
28	Trigonelline	9.1, 8.8, 8.8, 8.1, 4.4	Singlet, Doublet, Doublet, Triplet, Singlet	0.032 (0.015 / 100)	0.021 (0.007 / 87.5)	0.016 (0.007 / 87.5)	0.027 (0.014 / 100)	0.020 (0.007 / 87.5)
29	Trimethylamine N-oxide	3.3	Singlet	0.167 (0.154 / 37.5)	0.021 (0.018 / 50)	0.011 (0.006 / 75)	0.125 (0.139 / 62.5)	0.013 (0.016 / 87.5)
30	Tryptophan	10.2, 7.7, 7.5, 7.3, 7.3, 7.2, 4.1, 3.5, 3.3	Singlet, Doublet, Doublet, Singlet, Triplet, Triplet, Quartet, Quartet, Quartet	0.019 (0.006 / 87.5)	0.012 (0.003 / 87.5)	0.013 (0.004 / 75)	0.018 (0.005 / 87.5)	0.013 (0.004 / 100)
31	Tyrosine	7.2, 6.9, 3.9, 3.2, 3.0	Doublet, Doublet, Quartet, Quartet, Quartet	0.085 (0.024 / 50)	0.039 (0.014 / 75)	0.040 (0.010 / 62.5)	0.082 (0.024 / 62.5)	0.041 (0.012 / 62.5)
32	Valerate	2.1, 1.5, 1.3, 0.9	Triplet, Multiplet, Multiplet, Triplet	- (- / 0)	- (- / 0)	- (- / 0)	- (- / 0)	0.042 (0.000 / 12.5)
33	Valine	3.6, 2.3, 1.0, 1.0	Doublet, Multiplet, Doublet, Doublet	0.107 (0.048 / 100)	0.046 (0.018 / 100)	0.039 (0.014 / 87.5)	0.107 (0.048 / 100)	0.047 (0.019 / 100)
